# Upcycling Waste Expanded Polystyrene into Fe@Graphitic-Carbon Catalysts for Glycolytic Recycling of PET to BHET

**DOI:** 10.3390/polym18161983

**Published:** 2026-08-14

**Authors:** Jong In Choi, Chitra Sarkar, Yujin Kang, Saira Kanwal, Youn-Sang Bae, Do-Young Hong

**Affiliations:** 1 Research Center for Nanocatalysts, Korea Research Institute of Chemical Technology (KRICT), 141 Gajeong-ro, Yuseong, Daejeon 34114, Republic of Korea; xk1423@krict.re.kr (J.I.C.); chitra@krict.re.kr (C.S.); yujink@krict.re.kr (Y.K.); saira@krict.re.kr (S.K.); 2 Department of Chemical and Biomolecular Engineering, Yonsei University, 50 Yonsei-ro, Seodaemun-gu, Seoul 03722, Republic of Korea; 3 Department of Advanced Materials and Chemical Engineering, University of Science and Technology (UST), Yuseong-gu, Daejeon 34113, Republic of Korea

**Keywords:** BHET, chemical recycling, expanded polystyrene, Fe@graphitic-carbon catalyst, PET glycolysis

## Abstract

Chemical glycolysis can convert waste poly(ethylene terephthalate) (PET) into bis(2-hydroxyethyl) terephthalate (BHET), but recyclable heterogeneous catalysts derived from circular carbon sources and showing low metal release remain limited. Here, post-consumer expanded polystyrene (EPS) was transformed into a hypercrosslinked polymer (HCP) and carbonized with Fe to produce Fe@graphitic-carbon (Fe@C) catalysts for PET glycolysis. The catalysts are denoted *m*Fe@EPS-HCP_800_, where *m* represents the nominal Fe loading (wt.%), and 800 is the carbonization temperature (°C). The optimized *5*Fe@EPS-HCP_800_ contained graphitic carbon layers, bamboo-like carbon nanotube (CNT) domains, hierarchical porosity, and Fe-rich domains associated with graphitic carbon coverage. Under atmospheric-pressure conditions (PET, 2.00 g; ethylene glycol, 20.00 g; catalyst, 0.20 g; 200 °C; 2 h), it achieved complete PET conversion and 94.6% BHET yield. The catalyst also maintained BHET yields of ca. 90–94% over ten reuse runs, and post-reaction microscopy confirmed the retention of graphitic carbon layers and Fe-containing domains. Fe was below the detection limit in the product solutions for the 1, 3, and 5 wt.% Fe catalysts, whereas *7*Fe@EPS-HCP_800_ released 9.1 mg kg^−1^ Fe, consistent with incomplete carbon coverage at excessive Fe loading. Conversion profiles followed an Avrami–Erofeev/Weibull model, giving an apparent activation energy of 205.6 kJ mol^−1^. The data support a two-stage pathway in which external graphitic carbon/CNT domains promote primary PET chain scission to soluble oligomers, followed by Fe@C interfacial secondary glycolysis to BHET. This work demonstrates dual waste-polymer valorization by using EPS waste as catalytic infrastructure for PET chemical recycling.

## 1. Introduction

The accumulation of plastic waste remains a materials-management problem because most commodity polymers are designed for durability rather than circular end-of-life recovery. Mechanical recycling can retain material value when the waste stream is clean and compositionally uniform, yet repeated melt processing, additives, colorants, and mixed-polymer contamination often reduce product quality. Chemical recycling addresses this limitation by converting polymers back to monomers or chemically useful intermediates, thereby decoupling product quality from the thermal and contamination history of the waste stream [1,2,3,4,5,6,7,8].

Poly(ethylene terephthalate) (PET) is a major target for chemical recycling because it is widely used in beverage bottles, packaging, films, and polyester fibers. The ester backbone of PET enables solvolytic depolymerization by glycolysis, methanolysis, hydrolysis, and aminolysis. Among these routes, glycolysis is especially attractive for closed-loop polyester manufacturing because ethylene glycol (EG) cleaves PET to bis(2-hydroxyethyl) terephthalate (BHET), a purified monomer that can be repolymerized to PET or used in other polyester and polyurethane formulations [5,6,7,8,9,10,11]. In practice, however, uncatalyzed glycolysis is slow and typically requires high EG loading, high temperature, and long reaction time. The catalyst must therefore accelerate ester bond cleavage while suppressing excessive oligomer formation, metal contamination, and catalyst loss.

Metal salts, ionic liquids, deep eutectic solvent (DES) systems, organic bases, and pseudo-homogeneous nanoparticles can promote PET glycolysis with high conversion and high BHET yield [9,12,13,14,15,16,17,18]. DES systems are particularly useful benchmarks because they can be prepared from simple components and can achieve high BHET yields under relatively mild conditions [15,16]. For process implementation, solid heterogeneous catalysts remain important because they simplify catalyst recovery, product purification, and control of catalyst-derived residues. Carbon-supported metal catalysts are particularly relevant because metal sites can activate the ester carbonyl, while graphitic carbon domains provide thermal conductivity, dispersion of metal species, and adsorption environments for aromatic PET fragments. The reported performance of Fe_3_O_4_-boosted multiwalled carbon nanotubes (MWCNTs) and magnetic hollow ZnO-Fe_3_O_4_ micro/nanoaggregates illustrates the importance of combining Lewis-acidic metal centers with accessible carbon or oxide interfaces [19,20].

A second design requirement is the origin and lifetime of the catalyst. Waste-derived catalysts based on orange peel ash, bamboo leaf ash, oyster shells, ostrich eggshells, and PET-bottle labels have been explored as low-cost alternatives [18,21,22,23,24,25,26]. These studies demonstrate that waste resources can provide catalytically active inorganic phases, but ash-based materials often offer limited control over pore architecture, metal dispersion, and site accessibility. Expanded polystyrene (EPS) represents a different type of waste resource. EPS foam is bulky, aromatic, low in heteroatom content, and difficult to compact, but its phenyl-rich backbone is a suitable precursor for porous organic networks and carbon materials [27,28,29,30]. Direct pyrolysis of EPS is inefficient because the linear polymer volatilizes and collapses before a robust carbon framework develops. Hypercrosslinking can overcome this limitation by converting EPS into a rigid, porous aromatic network before carbonization.

Here, we report an EPS-derived Fe@graphitic-carbon (Fe@C) catalyst for PET glycolysis. The strategy links two waste-polymer streams by converting EPS into a catalyst precursor and PET into BHET. The catalyst is prepared through Friedel–Crafts hypercrosslinking of EPS, followed by Fe impregnation and controlled carbonization. The structural, catalytic, and kinetic results suggest a hierarchical Fe@C architecture in which graphitic carbon/CNT external domains assist primary PET chain fragmentation, while pore-mouth Fe@C interfacial sites are proposed to promote secondary glycolysis of soluble fragments. This mechanistic description is treated as a plausible interpretation rather than direct proof of every elementary step. The optimized *5*Fe@EPS-HCP_800_ catalyst combines accessible mesoporosity, ultramicroporous graphitic-shell features, graphitic ordering, Fe-rich domains associated with graphitic carbon coverage, and bamboo-like CNT domains. The manuscript therefore emphasizes the structure–activity relationship, kinetic behavior, regeneration stability, and mechanistic implications relevant to recycling and reuse of waste polymers [7,27,28,29,30,31,32,33].

## 2. Materials and Methods

### 2.1. Materials

Post-consumer EPS packaging foam was collected locally and used as the carbon precursor. Commercial PET bottles were washed with water, dried, and cut into approximately 5 mm × 5 mm flakes before glycolysis. Ethylene glycol (EG), 1,2-dichloroethane, formaldehyde dimethyl acetal, iron(III) chloride (FeCl_3_), iron(III) acetylacetonate [Fe(acac)_3_], dichloromethane, graphene oxide, amorphous carbon, and multi-walled carbon nanotubes (MWCNTs) were purchased from Sigma-Aldrich (St. Louis, MO, USA). Acetone, ethanol, methanol, tetrahydrofuran (THF), and acetonitrile were purchased from DUKSAN PURE CHEMICALS Co., Ltd. (Ansan, Gyeonggi-do, Republic of Korea). Unless otherwise noted, all reagents were used as received without further purification. were used as received unless otherwise stated. Directly weighed masses are reported according to the readability of the balance used. Gram-scale masses used in glycolysis tests are reported to 0.01 g, and smaller precursor masses were measured using an analytical balance when required.

### 2.2. Synthesis of the EPS-Derived Hypercrosslinked Polymer

Waste EPS foam (10.00 g) was dissolved in acetone (100 mL), dried at 100 °C overnight to remove solvent, compacted into an EPS ingot, and ground into powder. The EPS-derived hypercrosslinked polymer (as-EPS-HCP) was synthesized by Friedel-Crafts crosslinking. EPS powder (10.00 g), formaldehyde dimethyl acetal (27 mL), FeCl_3_ (46.00 g), and 1,2-dichloroethane (150 mL) were placed in a 500 mL two-neck flask equipped with a mechanical stirrer. The mixture was purged with N_2_ for 60 min and stirred until homogeneous. The reaction was then heated to 80 °C and maintained for 18 h. After cooling, the solid was washed repeatedly with ethanol/water (5:1, *v*/*v*) until the filtrate reached near-neutral pH. To remove residual soluble or weakly bound Fe species introduced by FeCl_3_ during the Friedel–Crafts hypercrosslinking step, the solid was refluxed in methanol (100 mL) for 1 h using an oil bath set to 100 °C. After cooling to room temperature, the suspension was vacuum-filtered, and the recovered solid was washed with fresh methanol several times. The washed solid was then dried at 100 °C overnight. Centrifugation was not used in this purification step.

### 2.3. Preparation of Fe/Carbon Catalysts

Carbonized EPS-HCP supports were prepared by heating as-EPS-HCP under Ar at 700, 800, or 900 °C for 3 h with a heating rate of 2 °C min^−1^. The products are denoted EPS-HCP_T_, where T is the carbonization temperature. Fe-loaded catalysts were prepared by impregnation with Fe(acac)_3_ followed by carbonization. In a typical synthesis, Fe(acac)_3_ was dissolved in ethanol (25 mL), as-EPS-HCP (2.00 g) was added, and the suspension was dried at 80 °C for 12 h. The impregnated precursor was carbonized under Ar at 800 °C for 3 h. Catalysts are denoted *m*Fe@EPS-HCP_T_, where *m* is the nominal Fe loading (1, 3, 5, or 7 wt.%) and T is the carbonization temperature. The catalyst *5*Fe@EPS-HCP_800_ was selected for detailed PET glycolysis studies.

### 2.4. PET Glycolysis and Product Analysis

PET glycolysis was conducted in a 100 mL three-neck flask equipped with a thermometer, mechanical stirrer, and reflux condenser under atmospheric pressure. PET flakes, EG, and catalyst were introduced into the reactor and heated to the target temperature. Unless otherwise noted, the standard conditions were PET (2.00 g), EG (20.00 g), catalyst (0.20 g), 200 °C, and 2 h. After reaction, the mixture was cooled to approximately 120 °C and hot-filtered to separate the catalyst and unconverted PET. The solid fraction was washed with acetone and dried at 100 °C overnight. The liquid product was first diluted 100-fold with tetrahydrofuran on a mass basis by mixing 1 part of liquid product with 99 parts of tetrahydrofuran by weight. An aliquot of this primary diluted solution was then diluted 100-fold with acetonitrile on a volume basis. The overall dilution factor before HPLC analysis was therefore 10,000. HPLC analysis was performed using a Youngin YL9100 system (YOUNGIN Chromass, Anyang, Republic of Korea) equipped with a UV–vis detector (YOUNGIN Chromass, Anyang, Republic of Korea) operated at 254 nm and a ZORBAX Eclipse XDB-C18 column (Agilent Technologies, Santa Clara, CA, USA) (4.6 mm × 150 mm, 5 µm). The mobile phase was 70% acetonitrile, the flow rate was 0.35 mL min^−1^, and the column temperature was 35 °C.

PET conversion, BHET selectivity, and BHET yield were calculated using Equations (1)–(3):

(1) $$\mathrm{PET}\;\mathrm{conversion}\;\left(\%\right)\;=\;\frac{W_{\mathrm{PET},i}-W_{\mathrm{PET},u}}{W_{\mathrm{PET},i}}\;\times 100\%$$

(2) $$\mathrm{BHET}\;\mathrm{selectivity}\;(\%)=\frac{W_{\mathrm{BHET}}/M_{\mathrm{BHET}}}{W_{\mathrm{PET},d}/M_{\mathrm{PET},\mathrm{repeat}}}\;\times 100\%$$

(3) $$\mathrm{BHET}\;\mathrm{yield}\;\left(\%\right)\;=\frac{W_{\mathrm{BHET}}/M_{\mathrm{BHET}}}{W_{\mathrm{PET},i}/M_{\mathrm{PET},\mathrm{repeat}}}\;\times 100\%$$

where *W*_PET,*i*_ is the initial mass of PET, *W*_PET,*u*_ is the mass of unconverted PET, *W*_PET,*d*_ is the mass of depolymerized PET (*W*_PET,*i*_ − *W*_PET,*u*_), *W*_BHET_ is the mass of quantified BHET, *M*_BHET_ is 254 g mol^−1^, and *M*_PET,*repeat*_ is 192 g mol^−1^. Catalytic reactions were performed at least in duplicate, and error bars represent standard deviations of replicated reactions or repeated analytical measurements.

### 2.5. Catalyst Regeneration and Reuse

After each glycolysis run, the catalyst was separated by vacuum filtration, washed with acetone, treated with THF to remove adsorbed BHET and oligomeric residues, filtered, dried, and reused. The recovered catalyst was weighed before each reuse experiment. The reuse test was conducted as a single sequential ten-cycle series, where the recovered and regenerated catalyst from each run was used in the next run. Because physical catalyst loss during filtration was approximately 5%, the catalyst dosage was restored to the initial level by adding the required amount of fresh catalyst before reuse. The error bars in the recyclability data represent the standard deviation of repeated HPLC analyses of the same product sample, rather than independent reaction replicates.

### 2.6. Characterization

#### 2.6.1. Spectroscopic and Thermal Characterization

Fourier-transform infrared (FT-IR) spectra were recorded using a Bruker ALPHA II spectrometer (Bruker Optics GmbH & Co. KG, Ettlingen, Germany) over the range of 4000–400 cm^−1^ at a resolution of 4 cm^−1^ by averaging 64 scans. Solid-state ^13^C cross-polarization/magic-angle-spinning nuclear magnetic resonance (^13^C CP/MAS NMR) spectra were acquired using a Varian VNMRS 600 spectrometer (Varian, Inc., Palo Alto, CA, USA). Because the measurements were performed in the solid state, no solvent was used. Thermogravimetric analysis (TGA) was performed using a TA Instruments SDT Q600 (TA Instruments, New Castle, DE, USA) from 40 to 800 °C at a heating rate of 10 °C min^−1^ under flowing air at 50 mL min^−1^. Derivative thermogravimetric (DTG) curves were obtained from the corresponding TGA data.

#### 2.6.2. Structural and Textural Characterization

Powder X-ray diffraction (XRD) patterns were collected using a Malvern Panalytical diffractometer (Malvern Panalytical B.V., Almelo, The Netherlands) with Cu Kα radiation (40 kV, 30 mA, λ = 1.542 Å) over a 2theta range of 10–80° at a scan rate of 2° min^−1^. The apparent crystallite sizes of the metallic Fe-like domains were estimated from the corresponding Fe-related reflection using the Scherrer equation, D = Kλ/(β cosθ), where β is the full width at half maximum in radians and K is the shape factor of 0.9. Nitrogen adsorption–desorption isotherm of the as-prepared EPS-HCP precursor was obtained at 77 K using a Micromeritics TriStar 3000 (Micromeritics Instrument Corporation, Norcross, GA, USA). Ar adsorption–desorption isotherms of the carbonized catalysts were obtained at 87 K using a Micromeritics 3Flex (Micromeritics Instrument Corporation, Norcross, GA, USA). Before analysis, the samples were degassed at 150 °C for 12 h under vacuum. Specific surface areas were calculated using the Brunauer–Emmett–Teller (BET) method. Mesopore-size distributions were estimated using the Barrett–Joyner–Halenda (BJH) method from the desorption branch, whereas ultramicropore-size distributions were evaluated using the Horvath–Kawazoe (HK) method following established physisorption practice [33].

#### 2.6.3. Raman, Microscopy, and Surface Analysis

Raman spectra were collected using a Bruker Raman system (Bruker Optics GmbH & Co. KG, Ettlingen, Germany) equipped with a 532 nm excitation laser. After baseline correction, the spectra were deconvoluted into the D, G, D′, and D″ components following established interpretations for disordered and amorphous carbon [34,35]. The I_D_/I_G_ and I_D”_/I_G_ ratios were used as comparative descriptors of graphitic disorder and amorphous-carbon contribution, respectively. Scanning electron microscopy (SEM) images were acquired using a TESCAN Mira 3 LMU FEG microscope (TESCAN, Brno, Czech Republic). Transmission electron microscopy (TEM), high-resolution TEM (HRTEM), high-angle annular dark-field scanning TEM (HAADF-STEM), and energy-dispersive X-ray spectroscopy (EDS) analyses were performed using a FEI Tecnai G2 20 microscope (FEI Company, Hillsboro, OR, USA) equipped with a LaB6 electron source and operated at 200 kV. X-ray photoelectron spectroscopy (XPS) was performed using a Kratos AXIS Supra spectrometer (Kratos Analytical Ltd., Manchester, UK) with an Al Kα X-ray source operated at 15 kV and 15 mA. Binding energies were calibrated using the C 1s C–C/C=C component at 284.8 eV [36]. Temperature-programmed desorption (TPD) profiles were recorded using a Micromeritics AutoChem II 2920 instrument (Micromeritics Instrument Corporation, Norcross, GA, USA) equipped with a thermal conductivity detector. Samples for TPD analysis were pretreated at 500 °C for 1 h in He flow, followed by gas adsorption at 40 °C for 30 min.

#### 2.6.4. Fe Leaching Analysis

The Fe concentration in the post-reaction liquid product was determined by ICP-AES using a Thermo Scientific iCAP 6500 Duo instrument (Thermo Fisher Scientific, Cambridge, UK). After PET glycolysis, the reaction mixture was filtered while hot to remove the catalyst and any remaining solid PET or insoluble residues. An aliquot of the liquid product was acidified and diluted with nitric acid solution, and the Fe concentration was quantified using an external Fe calibration curve. The Fe concentration was reported in mg kg^−1^ relative to the liquid product, and values below the analytical detection limit of 1 mg kg^−1^ were reported as not detected.

## 3. Results and Discussion

### 3.1. Catalyst Design and Dual Waste-Polymer Valorization

**Scheme 1:**
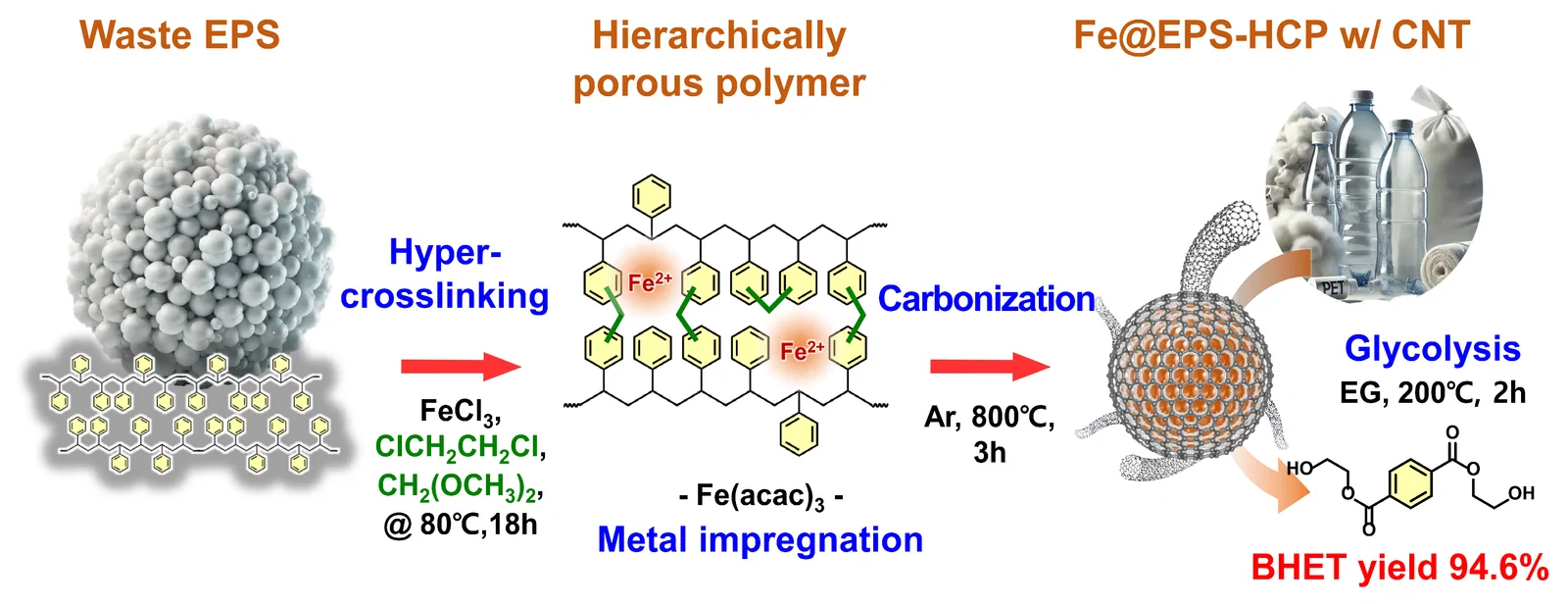
Upcycling of waste EPS into Fe@graphitic-carbon catalysts and their application in PET glycolysis to BHET. EPS is converted into a hypercrosslinked polymer through Friedel-Crafts crosslinking, followed by Fe impregnation and carbonization to form Fe@graphitic-carbon (Fe@C) catalysts for glycolytic PET recycling.

The overall concept is shown in Scheme 1. The preparation begins with waste EPS foam, which is converted into a hypercrosslinked porous polymer using formaldehyde dimethyl acetal as the external crosslinker and FeCl_3_ as the Friedel–Crafts catalyst. This step converts a soluble linear aromatic polymer into an insoluble porous network that can retain carbon yield and morphology during high-temperature treatment. Subsequent Fe impregnation and carbonization under Ar at 800 °C generate an Fe/graphitic carbon composite containing graphitized domains and carbon nanotube-like shells. The resulting catalyst is then used for PET glycolysis in EG at 200 °C, converting waste PET into BHET. The approach therefore uses one waste polymer as catalytic infrastructure for recycling a second waste polymer.

### 3.2. Formation of the EPS-Derived Hypercrosslinked Precursor

**Figure 1:**
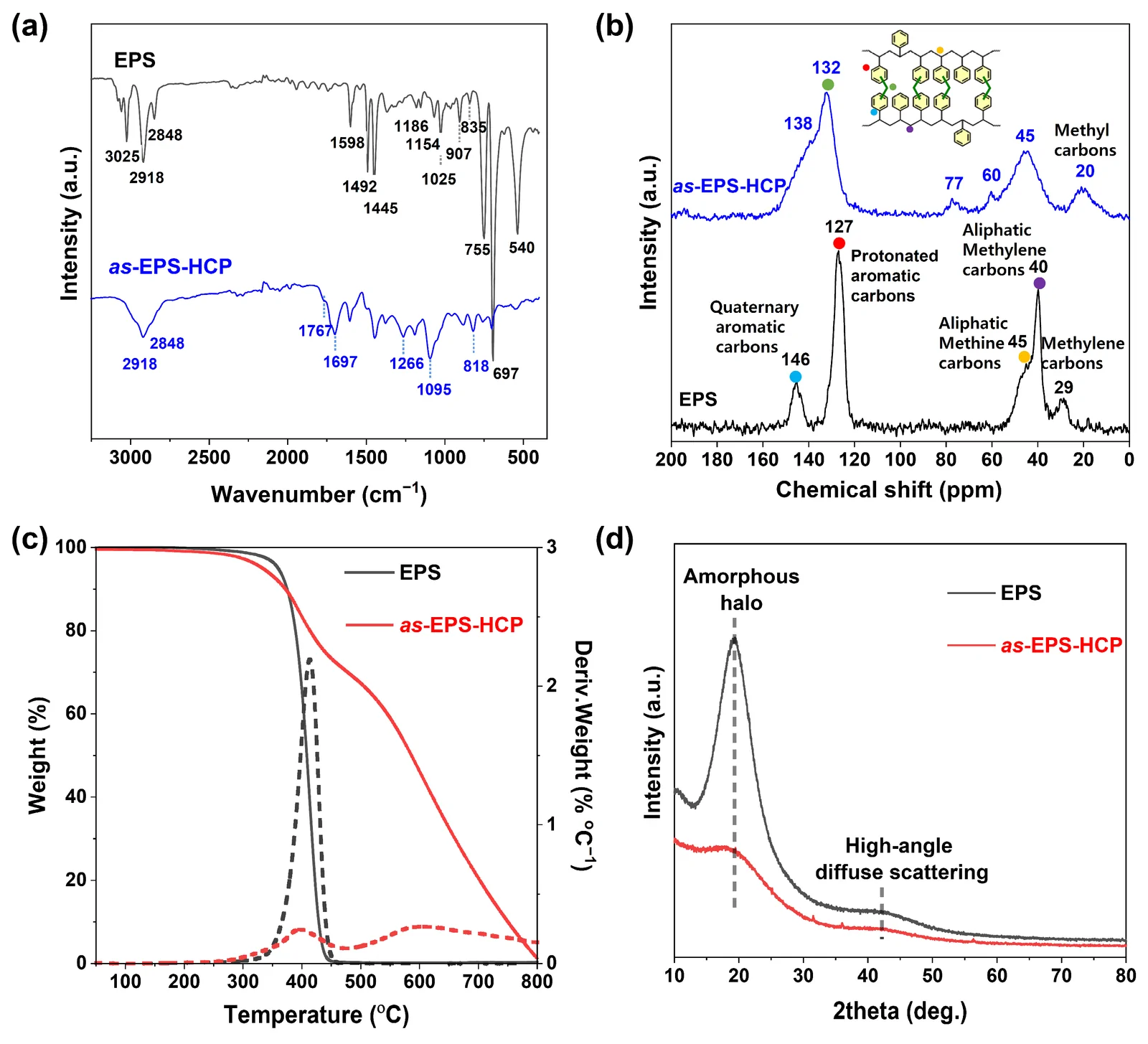
Characterization of pristine EPS and the EPS-derived hypercrosslinked precursor. (**a**) FT-IR spectra, (**b**) solid-state ^13^C CP/MAS NMR spectra, (**c**) TGA/DTG profiles, where the solid lines represent weight (%) and the dashed lines represent derivative weight (% °C^−1^) and (**d**) XRD patterns of pristine EPS and as-EPS-HCP.

The conversion of EPS into as-EPS-HCP was confirmed by FT-IR and solid-state ^13^C CP/MAS NMR spectroscopy (Figure 1a,b and Tables S1 and S2). In the FT-IR spectra, the out-of-plane C–H bending bands of monosubstituted benzene rings at 697 and 755 cm^−1^ decreased after hypercrosslinking, while the band near 818 cm^−1^ assigned to multi-substituted aromatic rings became more prominent. The attenuation of aromatic C–H stretching features and the emergence of additional bands associated with methylene and oxygen-containing linkages are consistent with electrophilic aromatic substitution and network formation. The ^13^C CP/MAS NMR spectrum of pristine EPS showed aromatic resonances near 146 and 127 ppm and aliphatic resonances near 45, 40, and 29 ppm. After hypercrosslinking, broadened aromatic signals and additional resonances associated with bridged and substituted aromatic environments appeared, indicating that the EPS chains were transformed into a chemically heterogeneous crosslinked network (Figure 1b and Figure S1a).

Thermal analysis further supports the formation of a crosslinked aromatic network (Figure 1c). Pristine EPS decomposed sharply near 400 °C, whereas as-EPS-HCP showed a broader mass-loss profile extending to higher temperature. This behavior is consistent with a constrained network in which chain mobility and volatilization are suppressed. XRD patterns showed that both EPS and as-EPS-HCP were largely amorphous, but as-EPS-HCP exhibited modified diffuse scattering associated with a more rigid porous aromatic framework (Figure 1d). The as-EPS-HCP precursor exhibited a BET surface area of 933.9 m^2^ g^−1^ and a total pore volume of 0.814 cm^3^ g^−1^ (Table 1), demonstrating that the hypercrosslinking converts bulky EPS foam into a high-surface-area carbon precursor. Hu et al. showed that waste EPS can be converted into porous organic polymers, and Gu et al. described hypercrosslinking as an effective route to rigid aromatic networks [27,29]. In line with these studies, the combined FT-IR, solid-state NMR, thermal, XRD, and sorption results indicate that EPS was converted into a porous hypercrosslinked aromatic framework suitable for Fe incorporation and carbonization.

### 3.3. Fe-Assisted Carbonization, Porosity, and Carbon Ordering

**Figure 2:**
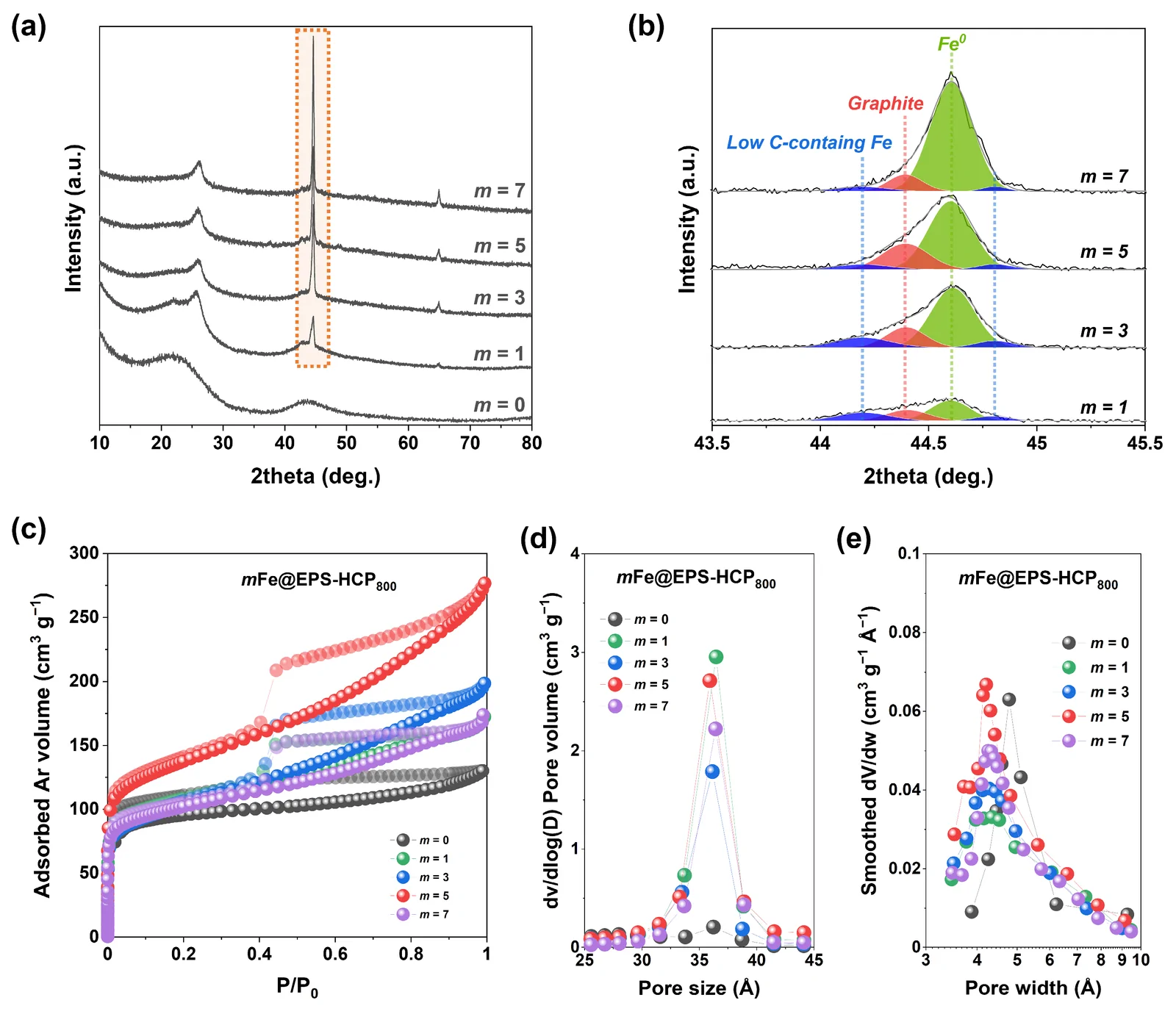
Structural evolution of Fe@graphitic-carbon catalysts. (**a**) XRD patterns of *m*Fe@EPS-HCP_800_ catalysts with nominal Fe loadings of 0–7 wt.%, (**b**) enlarged and deconvolution XRD profiles in the 43.5–45.5° region, (**c**) Ar adsorption–desorption isotherms, (**d**) mesopore size distribution calculated by the BJH method, and (**e**) micropore size distribution determined by the Horvath–Kawazoe method. The orange dashed box in (**a**) indicates the 43.5–45.5° region enlarged and deconvoluted in (**b**).

Carbonization of as-EPS-HCP changed both the pore system and the degree of carbon ordering. The XRD patterns of *m*Fe@EPS-HCP_800_ (Figure 2a) contain a broad carbon reflection in the 20–30° region and Fe-related features near 44–46°. Deconvolution of this region (Figure 2b and Figure S2) indicates overlapping contributions from graphitic carbon, low-carbon-containing Fe environments, and metallic Fe-like domains. Scherrer analysis of the metallic Fe-like diffraction feature gave apparent crystallite sizes of 20.6, 34.6, 34.9, and 61.7 nm for m = 1, 3, 5, and 7, respectively (Table S3). The modest change from 3 to 5 wt.% Fe and the sharp increase at 7 wt.% Fe indicate that the optimized catalyst contains Fe-containing domains associated with graphitized carbon, whereas excessive Fe loading promotes particle growth and agglomeration. Because metallic Fe, low- carbon-containing Fe domains, and Fe-C interfacial environments can exhibit closely overlapping diffraction features in the 44–46° region after Fe-assisted carbonization of aromatic carbon precursors, the XRD data are interpreted conservatively as evidence for Fe-containing crystalline domains embedded in graphitized carbon rather than as proof of a single isolated Fe phase [31,32,37].

The Ar physisorption results show that Fe loading does not simply block the pore system. A moderate Fe content improves accessible surface area and mesoporosity. The *5*Fe@EPS-HCP_800_ catalyst exhibited the highest BET surface area among the Fe-loaded samples (434.2 m^2^ g^−1^), with an external surface area of 204.9 m^2^ g^−1^ and a total pore volume of 0.353 cm^3^ g^−1^ (Table 1 and Figure 2c). These values were approximately 31%, 59%, and 60% higher, respectively, than those of *3*Fe@EPS-HCP_800_. This indicates that the 5 wt.% Fe promoted the most favorable development of accessible porosity. Relative to *5*Fe@EPS-HCP_800_, the 7 wt.% Fe catalyst showed decreases of approximately 23% in S_BET_, 45% in external surface area, and 37% in total pore volume, along with an increase in apparent Fe crystallite size from 34.9 to 61.7 nm. Thompson et al. and Hunter et al. reported that Fe species can promote carbon rearrangement and graphitization during carbonization [31,32]. The higher surface area and mesoporosity of *5*Fe@EPS-HCP_800_ are consistent with Fe-assisted carbon restructuring, whereas the lower textural values of *7*Fe@EPS-HCP_800_ indicate that excessive Fe loading promotes Fe-domain growth and partial pore occupation. The textural optimum at 5 wt.% Fe therefore reflects a balance between graphitic carbon formation and preservation of accessible porosity. HK analysis revealed an ultramicropore population centered at approximately 5.0–6.0 Å. Since HK-derived pore sizes are model-based accessible pore dimensions rather than direct structural spacings [33], this feature is assigned to defect-derived slit-like ultramicropores, pore-mouth nanogaps, and nanoscale fissures in the discontinuous or turbostratic graphitic shell and bamboo-like CNT walls generated during Fe-assisted carbonization [31,32,33]. These ultramicroporous features may enrich EG and provide local access to Fe@C interfacial environments after PET chains are first fragmented on external mesoporous graphitic surfaces.

### 3.4. Raman Evidence for Fe-Induced Graphitization and Defect Control

**Figure 3:**
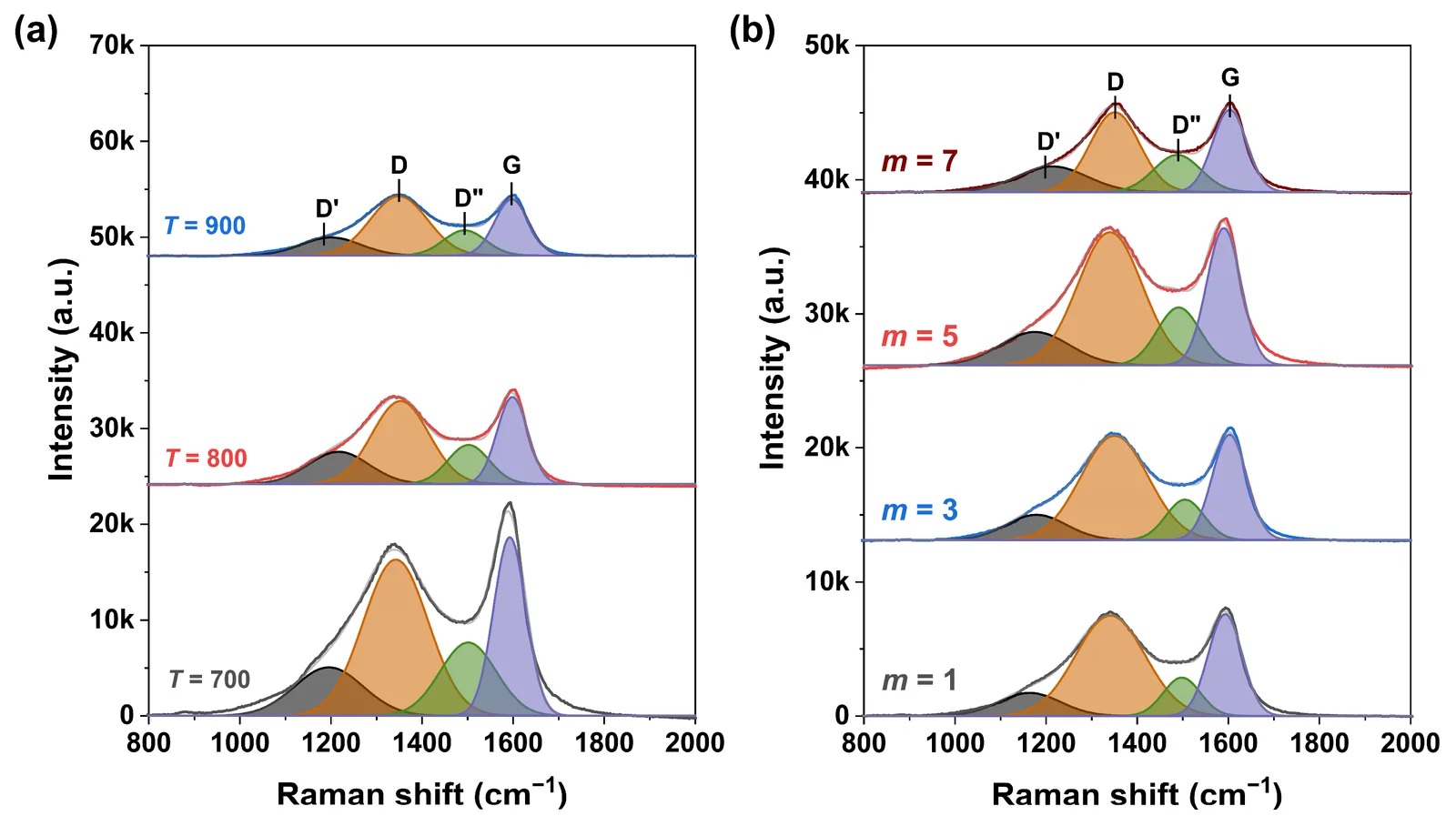
Deconvoluted Raman spectra of EPS-HCP-derived carbon materials. (**a**) Effect of carbonization temperature on the carbon structure of EPS-HCP-derived carbons. (**b**) Effect of Fe loading on the carbon ordering of *m*Fe@EPS-HCP_800_ catalysts. The D′, D, D″, and G bands were used to evaluate defect-rich graphitic domains, graphitic ordering, and amorphous carbon contributions. The I_D_/I_G_ and I_D″_/I_G_ ratios were compared to assess the degree of structural disorder and amorphous carbon contribution.

Raman spectroscopy provides a sensitive probe of the balance between graphitic order and defects (Figure 3). The spectra were deconvoluted into D, G, D′, and D″ bands. The G band corresponds to in-plane stretching of sp^2^ carbon, the D band is activated by defects in graphitic domains, D′ is associated with disorder in surface graphene layers, and D″ reflects amorphous sp^2^/sp^3^ carbon contributions [34,35]. For EPS-HCP carbons, increasing carbonization temperature decreased I_D_/I_G_ from 1.05 at 700 °C to 0.88 at 900 °C, consistent with progressive carbon ordering. However, excessively high carbonization temperature can reduce defect density and pore accessibility, whereas insufficient temperature leaves a more amorphous carbon matrix.

For *m*Fe@EPS-HCP_800_ catalysts, Fe loading promoted graphitization but also changed the defect distribution. The *5*Fe@EPS-HCP_800_ catalyst showed a relatively low I_D″_/I_G_ value (0.34), lower than those of the 1, 3, and 7 wt.% Fe samples, suggesting a reduced amorphous contribution and a more organized graphitic shell. At 7 wt.% Fe, the I_D″_/I_G_ value increased to 0.44, consistent with less uniform carbon reconstruction and Fe aggregation. Thus, the Raman data support the XRD and sorption results, indicating that a nominal Fe loading of 5 wt.% is sufficient to catalyze local graphitization while avoiding excessive particle growth and pore blockage. Following the assignments of Ferrari and Robertson and Sadezky et al., the D, G, D′, and D″ bands can be used to distinguish graphitic disorder, in-plane sp2 carbon, surface-layer defects, and amorphous carbon contributions in carbon materials [34,35]. On this basis, the low I_D″_/I_G_ value of *5*Fe@EPS-HCP_800_ supports the formation of a more ordered graphitic carbon framework with a reduced amorphous contribution.

### 3.5. Morphology and Graphitic Carbon Coverage of Fe-Containing Nanoparticles

**Figure 4:**
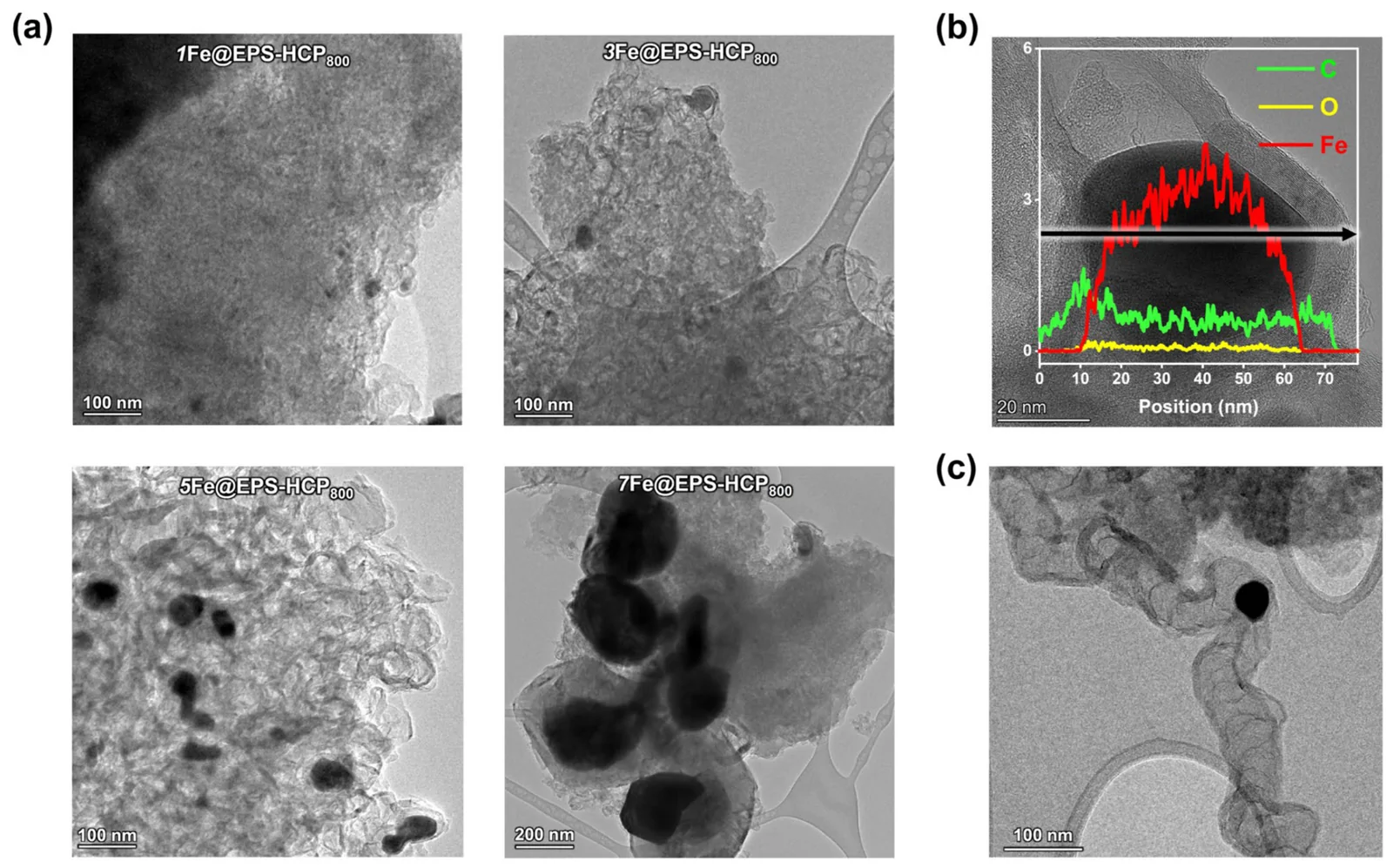
(**a**) TEM images of *m*Fe@EPS-HCP_800_ catalysts with different nominal Fe loadings. (**b**) Representative elemental line-scan profile of the *5*Fe@EPS-HCP_800_ catalyst superimposed on the corresponding high-resolution TEM image. The elemental line scan was acquired along the direction indicated by the arrow. (**c**) HRTEM image of the *5*Fe@EPS-HCP_800_ catalyst showing graphitic carbon layers around Fe-containing domains. Detailed HAADF-STEM/EDS line-scan profiles for 1, 3, 5, and 7 wt.% Fe catalysts are provided in Figure S4. The optimized *5*Fe@EPS-HCP_800_ sample shows graphitic-layer-associated Fe-containing nanoparticles and bamboo-like carbon nanotubes. The 7 wt.% Fe sample contains larger Fe-rich domains with less complete carbon coverage. Local graphitic-shell discontinuities are consistent with defect-derived ultramicropore and pore-mouth structures inferred from Ar physisorption.

TEM images show that Fe impregnation and carbonization changed the Fe domain distribution and the surrounding carbon morphology (Figure 4). At low Fe loading, the Fe-containing domains were sparse, whereas increasing the loading to 3–5 wt.% promoted the formation of graphitic carbon layers and bamboo-like CNT domains. The *5*Fe@EPS-HCP_800_ sample displayed a comparatively open, CNT-rich morphology, consistent with its increased external surface area and pore volume. The *7*Fe@EPS-HCP_800_ sample contained substantially larger Fe-rich particles, in agreement with the Scherrer-derived crystallite size increase to 61.7 nm and the measurable Fe leaching observed during glycolysis. This morphology explains why increasing nominal Fe loading beyond 5 wt.% did not improve catalytic performance.

HAADF-STEM/EDS line-scan profiles provide a comparative view of graphitic carbon coverage around Fe-rich domains as a function of metal loading (Figure 4 and Figure S4). For the 1, 3, and 5 wt.% Fe catalysts, the Fe signal is localized in the particle interior and is accompanied by carbon signals on both sides. This profile is consistent with Fe-rich domains surrounded by graphitic carbon layers in a core–shell-like Fe@C structure. The profile is most evident for *5*Fe@EPS-HCP_800_, where Fe-rich domains coexist with graphitic layers and bamboo-like CNTs. In contrast, the 7 wt.% Fe catalyst shows a broader Fe profile and an asymmetric line-scan response in which one side of the metal-rich domain is not accompanied by a carbon signal. This result suggests less complete carbon coverage of larger Fe-rich particles and is consistent with the Fe detected in the product solution for *7*Fe@EPS-HCP_800_. HRTEM further shows locally discontinuous graphitic fringes around Fe-containing domains, including edge openings and crack-like nanogaps. These features provide a structural basis for the HK-derived ultramicropores and pore-mouth defects, although HRTEM alone should not be treated as a quantitative measure of subnanometer pore width. Thus, *5*Fe@EPS-HCP_800_ is best described as a Fe@C catalyst with graphitic carbon coverage, bamboo-like CNT domains, and defect-rich pore mouths rather than as an exposed Fe or Fe oxide catalyst.

**Figure 5:**
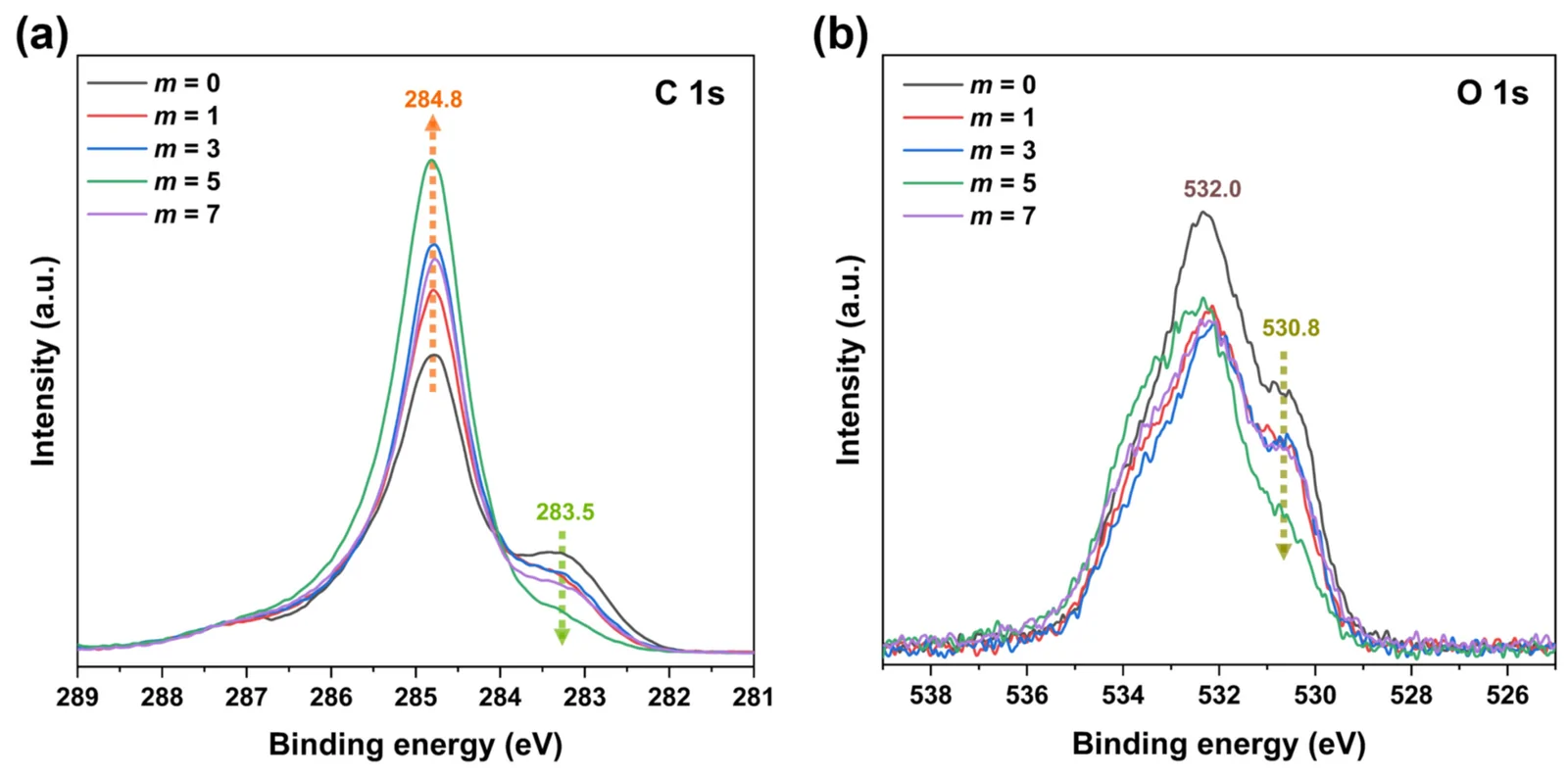
(**a**) C 1s and (**b**) O 1s XPS spectra of *m*Fe@EPS-HCP_800_ catalysts. The C 1s features near 283.5 and 284.8–285.0 eV, and the O 1s features near 530.8 and 532.0 eV are used to evaluate Fe-assisted graphitization, carbon-bound oxygen species, and graphitic carbon coverage around Fe-containing domains. The vertical dashed lines indicate the selected binding-energy positions, and the arrows are included as guides to highlight the corresponding spectral changes across the catalyst series.

The C 1s and O 1s XPS spectra provide further insight into the outer surface of the carbonized catalysts (Figure 5). The discussion is limited to reproducible peak-position and intensity trends in the C 1s and O 1s spectra. Oxygen-containing species remained detectable after carbonization, but this observation should not be interpreted as direct evidence for surface Fe oxide formation. Because XPS probes only the outer few nanometers of the catalyst, the O 1s signal can arise from carbon-bound oxygen species located at graphitic edges, CNT defects, pore mouths, residual oxygenated bridge fragments, and air-exposed carbon surfaces. This interpretation is consistent with the known sensitivity of carbon XPS to defect and oxygenated edge sites [34,35,36,38].

In the C 1s spectra, the feature near 283.5 eV decreased as the Fe loading increased, whereas the C–C/C=C envelope centered at approximately 284.8–285.0 eV became most pronounced for *5*Fe@EPS-HCP_800_. The low-binding-energy feature is tentatively associated with electron-rich carbon in contact with metallic Fe or Fe–C interfacial environments rather than Fe oxide. Its attenuation with increasing Fe content suggests that such interfacial carbon becomes less exposed to the outer surface as Fe-catalyzed carbon dissolution–precipitation produces graphitic shells around the Fe-containing domains. In contrast, the strengthened 284.8–285.0 eV contribution is consistent with the development of curved graphitic sp^2^ carbon, supporting the Raman, HRTEM, and HAADF line-scan evidence for graphitic carbon layers and bamboo-like CNT domains [31,32,34,35]. The maximum intensity of this graphitic-carbon envelope for *5*Fe@EPS-HCP_800_ is consistent with the optimized balance between Fe-assisted graphitization and preservation of accessible defect-rich carbon surfaces.

The O 1s spectra show a complementary trend. The feature centered near 530.8 eV decreased with increasing Fe loading. If surface-exposed Fe oxide were the dominant oxygen-containing phase, this low-binding-energy O 1s feature would be expected to increase with Fe content. The observed decrease instead indicates progressive removal, shielding, or burial of low-binding-energy carbon-bound oxygen species at defective graphitic edges during Fe-assisted graphitization. By contrast, the approximately 532.0 eV feature remained nearly unchanged over the Fe-loading series, indicating that it mainly reflects C–O/C–OH/C–O–C groups and/or weakly adsorbed oxygen- or water-derived species on the outer carbon shell rather than Fe–O species. The combined C 1s/O 1s peak trends therefore indicate a catalyst surface dominated by graphitic carbon/CNT shells with carbon-bound oxygen at defects and pore mouths, while the Fe-rich domains are mainly associated with graphitic carbon coverage rather than broadly exposed at the outer surface.

Surface acid and base properties were examined by CO_2_–TPD and NH_3_–TPD to clarify the role of surface functionality in PET glycolysis (Figure S7 and Table S4). The total basicity increased from 0.26 mmol g^−1^ for *1*Fe@EPS-HCP_800_ to 0.31 mmol g^−1^ for *5*Fe@EPS-HCP_800_, then decreased to 0.26 mmol g^−1^ for *7*Fe@EPS-HCP_800_. The maximum basicity of *5*Fe@EPS-HCP_800_ is consistent with the development of graphitic carbon layers and bamboo-like CNT domains. The extended conjugated π–electron network of graphitic carbon can provide electron-rich Lewis basic surface sites that assist EG activation, which agrees with reported Lewis base and basic carbon catalysts for PET glycolysis [13,15,16,17,18]. NH_3_–TPD showed that the acid site distribution also changed with Fe loading. As the Fe loading increased to 5 wt.%, the high-temperature desorption feature near 750 °C decreased, while the feature near 600 °C became more pronounced. This shift suggests that Fe-assisted graphitization reduced strongly adsorbing defect or oxygen-associated acid sites and increased accessible Fe@C interfacial acid sites. The lower 530.8 eV O 1s feature of *5*Fe@EPS-HCP_800_ is consistent with this reduced exposure of oxygen-rich defect sites. At 7 wt.% Fe, the 600 °C feature decreased, and the high-temperature feature increased again, which is consistent with incomplete graphitic encapsulation and partial exposure of Fe-rich domains. The TPD results therefore support the activity trend by showing that *5*Fe@EPS-HCP_800_ provides the most favorable balance between Lewis basic graphitic domains for EG activation and accessible Fe@C acid sites for ester carbonyl polarization.

### 3.6. Catalytic Glycolysis of PET and Fe-Loading Dependence

The catalytic role of the Fe@C architecture was first evaluated by comparing control carbon materials under identical glycolysis conditions (PET, 2.00 g; EG, 20.00 g; catalyst, 0.20 g; 200 °C; 2 h). Graphene oxide and amorphous carbon showed only limited PET conversion and low BHET yield, indicating that oxygenated or disordered carbon surfaces alone are insufficient to promote efficient glycolysis (Figure S5). EPS-HCP-derived carbons prepared without Fe also exhibited modest activity, even after carbonization at different temperatures, suggesting that the porous carbon scaffold by itself cannot provide enough carbonyl-activation sites for high-rate transesterification. In contrast, MWCNTs gave a substantially higher conversion and BHET yield than amorphous carbon and Fe-free EPS-HCP-derived carbons. This result indicates that graphitic carbon surfaces facilitate PET glycolysis, most likely by improving PET/oligomer adsorption, EG activation, and interfacial transesterification. However, the activity of MWCNTs remained lower than that of the optimized Fe-loaded EPS-HCP catalyst. This confirms that graphitic carbon is beneficial but not sufficient and that the cooperative Fe@C interfacial environment is required for rapid and selective BHET formation.

**Figure 6:**
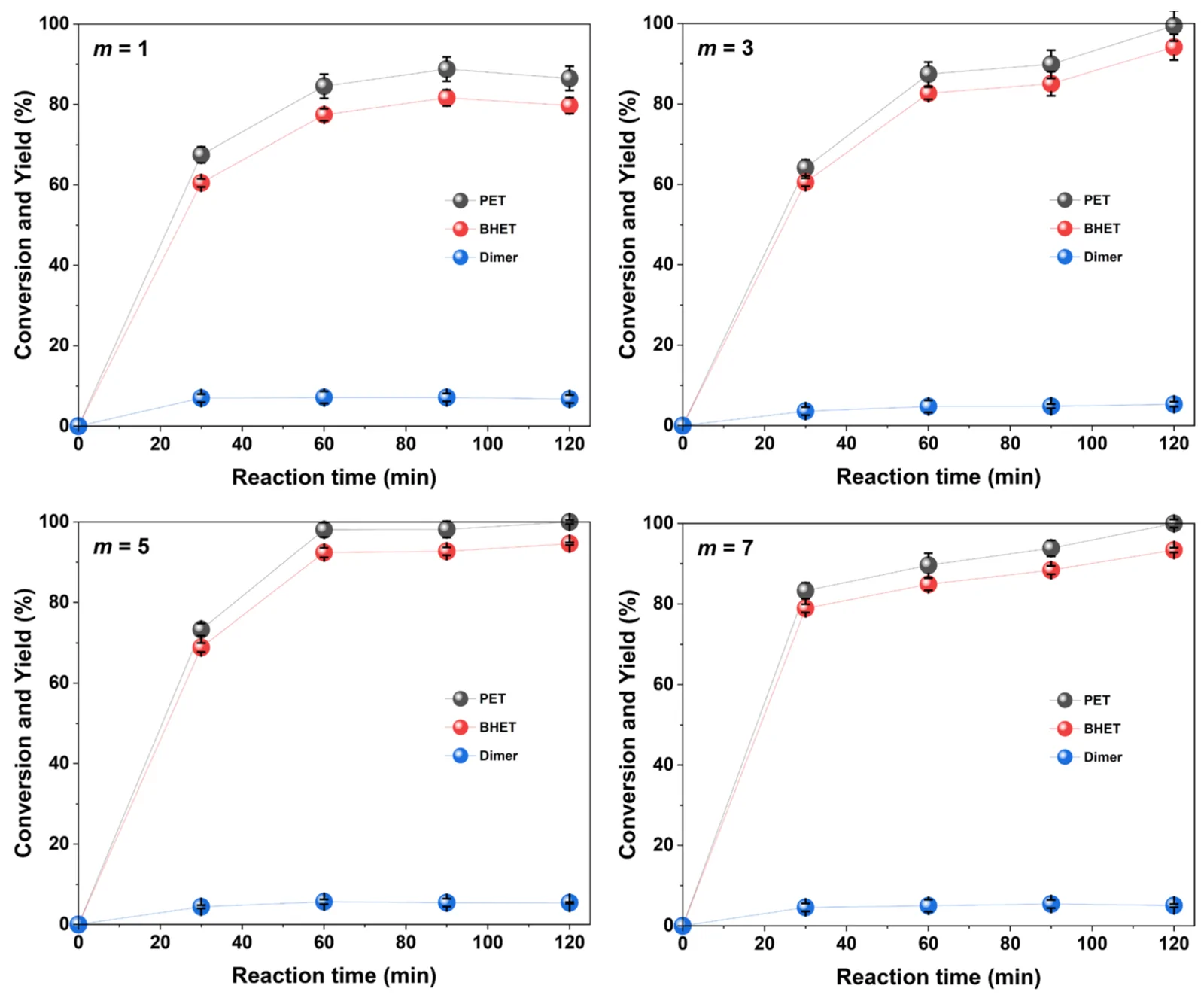
Fe-loading-dependent PET glycolysis over *m*Fe@EPS-HCP_800_ catalysts. Time profiles of PET conversion, BHET yield, and dimer yield are shown for catalysts with nominal Fe loadings of *m* = 1, 3, 5, and 7 wt.%. Reaction conditions: PET, 2.00 g; EG, 20.00 g; catalyst, 0.20 g; 200 °C; reaction time, 0–120 min. Error bars represent standard deviations of replicate experiments.

The Fe-loading dependence was then examined using *m*Fe@EPS-HCP_800_ catalysts as a function of reaction time (Figure 6). The *1*Fe@EPS-HCP_800_ catalyst showed slower PET conversion and lower BHET formation, which can be attributed to an insufficient density of Fe@C interfacial sites. Increasing the nominal Fe loading to 3 and 5 wt.% markedly accelerated PET glycolysis. In particular, *5*Fe@EPS-HCP_800_ achieved nearly complete PET conversion and a BHET yield of 94.6% after 120 min, while maintaining a low dimer yield. The catalytic trend was further compared with the textural properties in Table 1. The *5*Fe@EPS-HCP_800_ catalyst showed the highest BET surface area of 434.2 m^2^ g^−1^, external surface area of 204.9 m^2^ g^−1^, and total pore volume of 0.353 cm^3^ g^−1^, which coincided with the highest BHET yield. The developed mesoporosity likely improves PET wetting, oligomer diffusion, and access to Fe@C interfacial environments during the initial depolymerization stage. However, the activity cannot be described by BET surface area alone because Fe loading also changes graphitic carbon ordering, bamboo-like CNT formation, and graphitic carbon coverage around Fe-containing domains. The optimum activity of *5*Fe@EPS-HCP_800_ therefore reflects a balance between accessible porosity and Fe@C interfacial environments associated with graphitic carbon coverage. Further increasing the Fe loading to 7 wt.% did not meaningfully improve BHET yield, despite rapid initial PET conversion. The Fe concentration in the product solution was below the analytical detection limit of 1 mg kg^−1^ for *1*Fe@EPS-HCP_800_, *3*Fe@EPS-HCP_800_, and *5*Fe@EPS-HCP_800_, whereas *7*Fe@EPS-HCP_800_ showed measurable Fe leaching of 9.1 mg kg^−1^ under the same reaction and analytical conditions (Table S3). This behavior is consistent with the HAADF-STEM/EDS line-scan results in Figure S4. The 1–5 wt.% Fe catalysts show Fe-rich domains surrounded by carbon signals, consistent with graphitic carbon coverage around Fe-rich domains. In contrast, the 7 wt.% Fe catalyst contains metal-rich regions with incomplete carbon coverage on one side, indicating partial exposure of Fe domains. Such particles with less complete carbon coverage are more susceptible to leaching during glycolysis. Therefore, the superior performance of *5*Fe@EPS-HCP_800_ is associated with the combined development of accessible porosity, bamboo-like CNT structures, and Fe@C interfacial environments with graphitic carbon coverage.

### 3.7. Reaction Parameter Optimization

**Figure 7:**
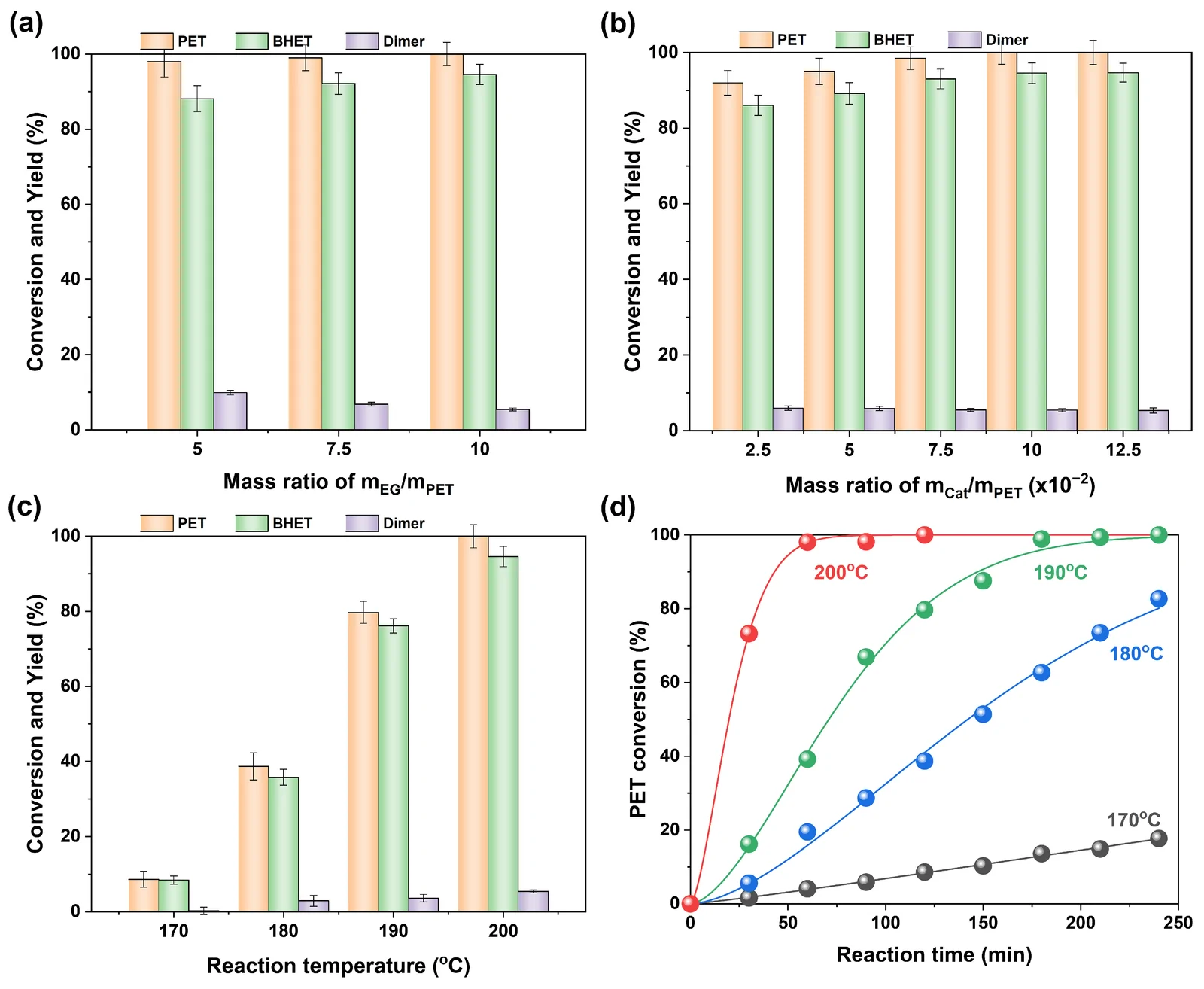
Optimization and temperature-dependent glycolysis behavior over *5*Fe@EPS-HCP_800_. Effects of (**a**) EG/PET mass ratio, (**b**) catalyst/PET mass ratio, and (**c**) reaction temperature on PET conversion, BHET yield, and dimer yield. (**d**) Time-dependent PET conversion at different reaction temperatures used for kinetic analysis. Unless otherwise specified, the standard reaction conditions were PET, 2.00 g; EG, 20.00 g; catalyst, 0.20 g; 200 °C; and 2 h. Error bars represent standard deviations of replicate experiments.

The reaction parameters for PET glycolysis over *5*Fe@EPS-HCP_800_ were optimized by varying the EG/PET mass ratio, catalyst dosage, and reaction temperature (Figure 7a–c). When the EG/PET mass ratio was increased from 5 to 10, PET conversion remained high under all conditions, whereas the BHET yield increased from approximately 88% to 94.6% (Figure 7a). This result indicates that excess EG mainly promotes the conversion of glycolyzed intermediates to BHET and shifts the transesterification equilibrium toward hydroxyethyl-terminated products. The improvement became less pronounced above an EG/PET ratio of 7.5, suggesting that a ratio of 10 is sufficient for efficient PET wetting and oligomer solubilization.

The catalyst dosage also influenced BHET formation (Figure 7b). Increasing the catalyst/PET mass ratio from 2.5 × 10^−2^ to 10 × 10^−2^ gradually improved both PET conversion and BHET yield. Further increasing the catalyst/PET ratio to 12.5 × 10^−2^ did not give a meaningful additional benefit. This plateau suggests that once enough Fe@C interfacial sites are available, the reaction is no longer limited only by the number of catalytic sites. PET softening, oligomer diffusion, and product dissolution also become important. Reaction temperature had the strongest effect on glycolysis performance (Figure 7c). At 170 °C, PET conversion and BHET yield were very low, indicating insufficient PET chain mobility and slow interfacial transesterification. The reaction became more effective at 180 °C and increased sharply between 190 and 200 °C. Nearly complete PET conversion and 94.6% BHET yield were obtained at 200 °C, with only a small amount of dimer. Therefore, the standard condition of EG/PET = 10, catalyst/PET = 10 × 10^−2^, 200 °C, and 2 h was selected for subsequent experiments.

### 3.8. Apparent Kinetic Analysis of PET Depolymerization over 5Fe@EPS-HCP_800_

The temperature-dependent conversion profiles in Figure 7d were analyzed to evaluate the apparent kinetics of PET glycolysis over *5*Fe@EPS-HCP_800_. Because the reaction proceeds in a solid–liquid system, the measured conversion does not represent only an elementary ester-exchange step. It also includes PET softening, EG penetration, chain-end exposure, oligomer dissolution, and interfacial glycolysis. The kinetic parameters should therefore be interpreted as apparent descriptors of the overall depolymerization process.

A pseudo-first-order (PFO) model was first considered because ethylene glycol was used in large excess relative to PET and served as the reaction medium. Under this condition, the ethylene glycol concentration can be approximated as nearly constant, allowing PET conversion to be treated as an apparent first-order process for comparison with previous PET glycolysis studies. The PFO model was therefore retained as a reference model rather than as the principal mechanistic description. The Avrami–Erofeev/Weibull model, expressed as X = 1 − exp[−(*k*_A_ t)*^n^*], provided a better representation of the full conversion-time behavior. As summarized in Table 2, the average R^2^ increased from 0.968 for the PFO model to 0.997 for the Avrami–Erofeev/Weibull model, while the average RMSE decreased from 0.044 to 0.012. These improvements indicate that the Avrami–Erofeev/Weibull model better describes the conversion behavior of the heterogeneous depolymerization system than the simple PFO model. The exponent *n* was higher than unity at all temperatures, supporting a distributed apparent kinetic behavior rather than a simple homogeneous first-order reaction. The fitted *k*_A_ increased systematically with temperature, from 9.94 × 10^−4^ min^−1^ at 170 °C to 3.99 × 10^−2^ min^−1^ at 200 °C. This trend is consistent with Figure 7d, where PET conversion was slow at 170 and 180 °C but became much faster at 190 and 200 °C. The sharp improvement at higher temperatures indicates that PET chain mobility and accessibility to catalytic interfacial sites are important factors in addition to chemical transesterification.

Arrhenius analysis gave an apparent activation energy (E_a_) of 205.6 kJ mol^−1^ from the Avrami–Erofeev/Weibull rate constants and 229.6 kJ mol^−1^ from the PFO rate constants (Table 3 and Figure S6). Although the PFO rate constants gave a slightly higher R^2^ in the Arrhenius plot, this value reflects only the linearity of ln *k* versus 1/T. It does not determine whether the model accurately describes the conversion-time data at each temperature. Accordingly, the Avrami-derived rate constants were used for the main kinetic interpretation because the model provided a more accurate fit to the full conversion profiles. The PFO-derived values were retained only for comparison with conventional PET glycolysis kinetics.

The kinetic results are consistent with the proposed two-regime depolymerization pathway. The initial conversion is governed by the accessibility and fragmentation of PET chains on graphitic carbon and CNT-rich external surfaces. After fragmentation, soluble oligomers or chain-end groups can participate in secondary glycolysis at pore-mouth and Fe@C interfacial environments. The Avrami–Erofeev/Weibull model does not prove a specific microscopic pathway, but it better captures the distributed reaction behavior expected for this heterogeneous depolymerization system [39,40,41,42,43].

### 3.9. Catalyst Reuse, Structural Retention, and Metal-Leaching Resistance

**Figure 8:**
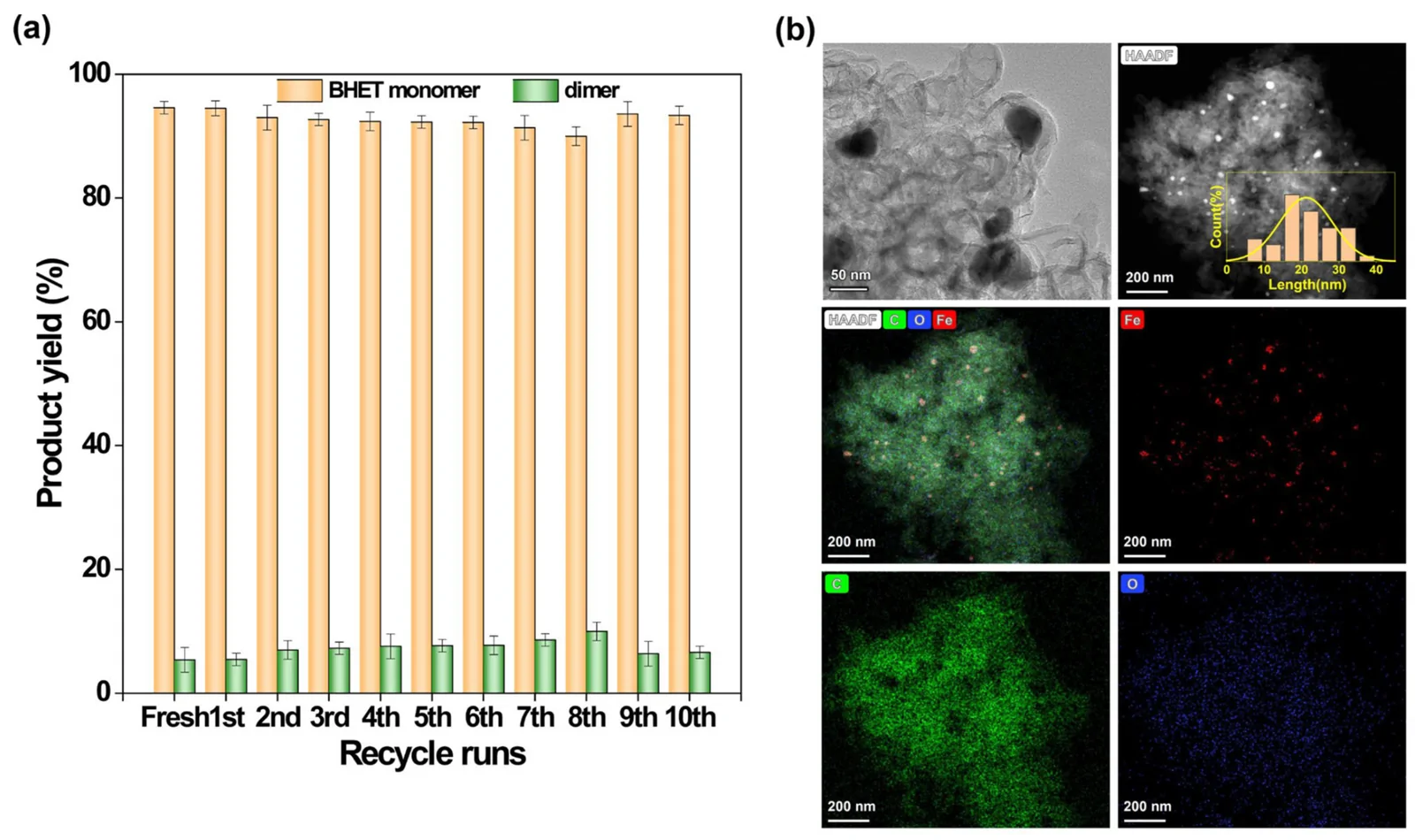
Recyclability and post-reaction structural characterization of *5*Fe@EPS-HCP_800_. (**a**) BHET monomer and dimer yields over ten reuse runs and (**b**) representative post-reaction HRTEM, HAADF-STEM image, and EDS elemental mapping of the catalyst after repeated glycolysis. The microscopy data show retention of graphitic carbon layers around Fe-rich domains, which is consistent with retention of the Fe@graphitic-carbon (Fe@C) architecture during reuse. Reaction conditions: PET, 2.00 g; EG, 20.00 g; catalyst, 0.20 g; 200 °C; reaction time, 2 h. Error bars represent standard deviations of repeated HPLC analyses of the product solution obtained from each recycle run.

The operational stability of *5*Fe@EPS-HCP_800_ was examined over ten consecutive PET glycolysis runs as shown in Figure 8a. The BHET monomer yield was maintained at ca. 90–94%, and dimer formation remained low throughout the reuse test. This stable product distribution indicates that the catalytic function of the Fe@C architecture was preserved during repeated reaction and regeneration. ICP-AES analysis showed that the Fe concentration in the product solution remained below the analytical detection limit (<1 mg kg^−1^) after both the first and tenth runs of the sequential reuse test. Post-reaction microscopy provides structural evidence for this stability, as shown in Figure 8b. The HAADF-STEM image shows Fe-rich domains still dispersed within the carbon matrix, while elemental mapping confirms their spatial association with the C-rich framework. The HRTEM image further shows that the graphitic carbon layers and CNT-like domains were retained around the Fe-containing regions after reuse. These observations suggest that the Fe-containing domains remained associated with the surrounding graphitic carbon framework during repeated glycolysis. The absence of detectable Fe in the product solution, together with the retained post-reaction Fe@C morphology, is consistent with a role of graphitic carbon coverage and CNT-rich domains in limiting metal release under the tested conditions. The high recyclability of *5*Fe@EPS-HCP_800_ is therefore associated with retained Fe-containing domains and the structural integrity of the surrounding carbon framework.

### 3.10. Proposed Catalytic Role of Fe/Carbon Interfaces and Benchmarking

**Figure 9:**
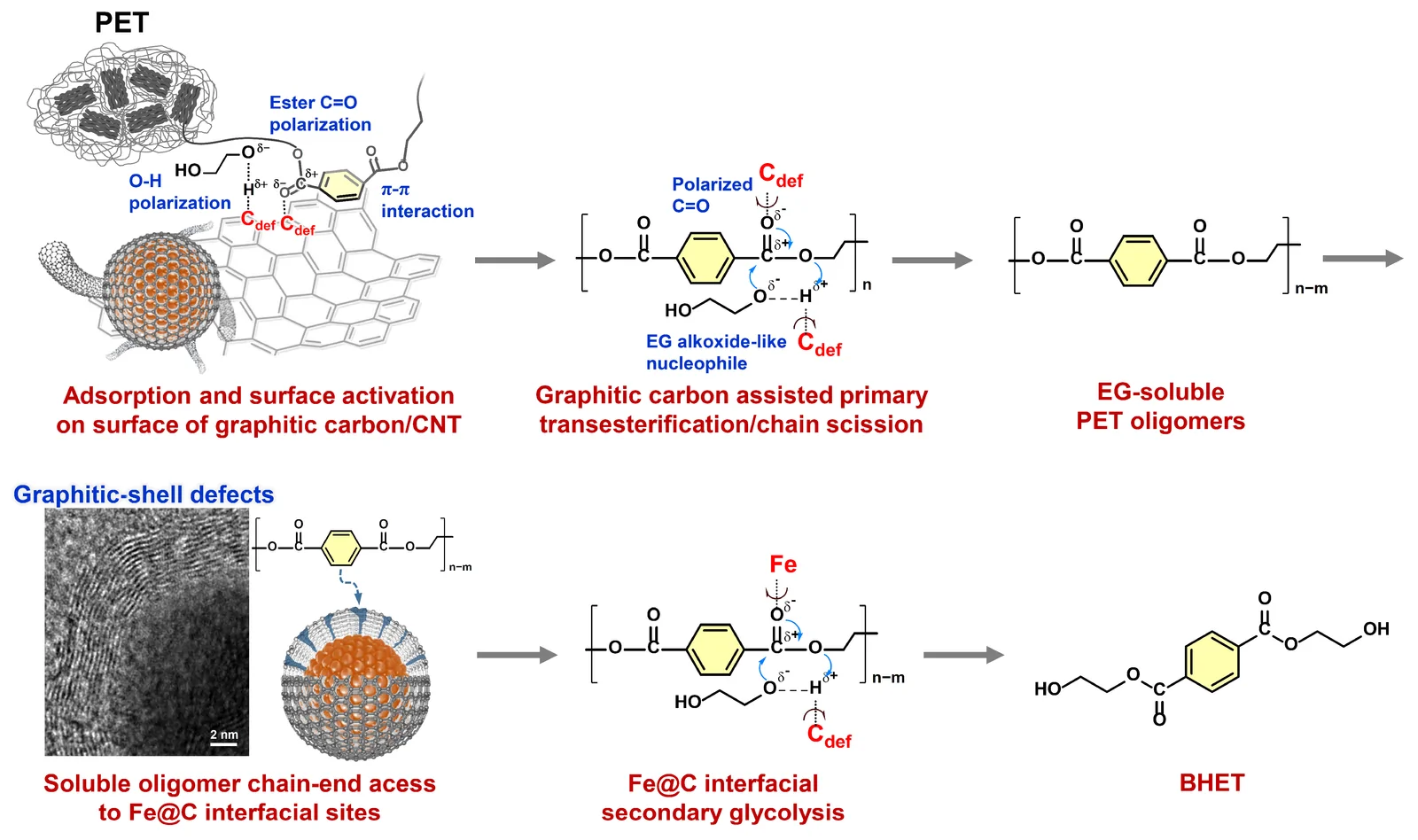
Plausible mechanism for PET glycolysis over *5*Fe@EPS-HCP_800_. External graphitic carbon/CNT domains promote PET adsorption, EG activation, and primary chain fragmentation to EG-soluble oligomers. Oligomer chain ends subsequently access pore-mouth Fe@graphitic-carbon (Fe@C) interfacial sites, where transient Fe···O=C interaction polarizes ester carbonyl groups and adjacent graphitic carbon assists EG nucleophilic attack, leading to secondary glycolysis and BHET formation.

A plausible reaction mechanism is proposed in Figure 9. Long PET chains are first adsorbed on external graphitic carbon layers and bamboo-like CNT domains through π–π interactions with terephthalate units and ester C=O polarization. Defect and edge sites in the sp^2^ carbon framework assist O–H polarization of EG, producing EG alkoxide-like nucleophilic character without requiring a freely dissolved strong alkoxide. This activated EG attacks the polarized PET ester carbonyl, causing primary transesterification and chain scission to form EG-soluble PET oligomers. These fragments then access pore-mouth Fe@graphitic-carbon (Fe@C) interfacial sites. At these confined interfaces, metallic Fe within the graphitic shell is proposed to polarize ester carbonyl oxygen through a transient Fe···O=C interaction, while adjacent graphitic carbon assists EG nucleophilic attack. This cooperative graphitic-carbon/Fe@C mechanism is consistent with the control reactions, microscopy, leaching behavior, and kinetic data. It should be regarded as a proposed mechanism because direct operando spectroscopy of the elementary steps was not performed [7,19,20,31,32,37,44]. Previous PET glycolysis studies provide a basis for this working model. Campanelli et al. treated glycolysis as progressive depolymerization with chain scission and conversion of PET-derived fragments [44]. Other kinetic studies showed that PET accessibility, swelling, and oligomer conversion affect apparent glycolysis rates [10,11]. Al-Sabagh et al. and Yun et al. reported that carbon frameworks combined with Fe-containing or oxide interfaces improve PET glycolysis in Fe_3_O_4_/MWCNT and hollow ZnO Fe_3_O_4_ systems [19,20]. These reports support the interpretation that graphitic carbon and CNT domains assist initial fragmentation, while Fe@C interfacial environments promote further conversion of soluble fragments to BHET. This remains a working model based on the combined structural, catalytic, and kinetic results.

Table 4 compares *5*Fe@EPS-HCP_800_ with representative deep eutectic solvent systems and heterogeneous catalysts for PET glycolysis. The urea/ZnCl_2_ and choline chloride/zinc acetate systems gave BHET yields of 82.8% and 91.6%, respectively, under relatively mild conditions [15,16], confirming that deep eutectic solvent catalysis is an effective benchmark for rapid PET glycolysis. The comparison therefore highlights complementary process features rather than a simple yield ranking. Deep eutectic solvent systems offer mild reaction conditions and high monomer yield, but separation of the catalytic liquid phase from BHET and ethylene glycol and its recovery for repeated use remain important process factors. In contrast, *5*Fe@EPS-HCP_800_ is a recoverable solid catalyst prepared from waste EPS. It provides 94.6% BHET yield, Fe concentration below the analytical detection limit at the optimized Fe loading and stable performance over ten consecutive catalyst reuse runs.

The comparison in Table 4 also points to process considerations that are relevant to PET glycolysis beyond conversion and BHET yield. PET-to-BHET recycling is most valuable when the recovered monomer can be repolymerized into high-purity PET for water bottles, beverage bottles, and food contact containers, where metal residues and other impurities must be strictly controlled [3,4,7]. In this regard, the graphitic carbon shell around the Fe-rich domains is important because the optimized catalyst showed no detectable Fe release into the product solution. Low metal release can reduce the need for repeated water washing of BHET before repolymerization, which would otherwise increase wastewater treatment and purification cost. When PET conversion is complete and byproduct formation is limited, BHET and EG separation can be simplified through low-temperature recrystallization or evaporation and recovery of EG [14]. The use of waste EPS as the carbon source can also improve material economy by converting a low-value polymer waste into catalytic infrastructure for PET recycling.

## 4. Conclusions

A waste-EPS-derived Fe@graphitic-carbon catalyst was developed for the glycolytic recycling of PET to BHET. Hypercrosslinking converted post-consumer EPS into an aromatic porous precursor, and Fe-assisted carbonization generated a hierarchical carbon architecture containing graphitic shells, bamboo-like CNT domains, ultramicroporous defects, and Fe-containing core–shell structures. Among the prepared catalysts, *5*Fe@EPS-HCP_800_ provided the best balance between structure and catalytic function, achieving complete PET conversion and 94.6% BHET yield at 200 °C within 2 h under reflux conditions. The results show that catalytic performance is governed not by Fe content alone, but by the balance between Fe incorporation and graphitic carbon coverage. Moderate Fe loading favored Fe-rich domains associated with the carbon framework, whereas excessive Fe loading led to incomplete carbon coverage and detectable Fe release. The reuse test, post-reaction microscopy, and HAADF-STEM/EDS results support structural retention of the optimized Fe@graphitic-carbon (Fe@C) architecture under the tested conditions.

Kinetic analysis indicated that PET glycolysis over *5*Fe@EPS-HCP_800_ is better described as an apparent heterogeneous depolymerization process than as a simple first-order reaction. The Avrami-Erofeev/Weibull model captured the temperature-dependent conversion profiles more accurately and gave an apparent activation energy of 205.6 kJ mol^−1^. This behavior is consistent with a two-regime pathway in which graphitic carbon and CNT-rich external surfaces assist initial PET chain fragmentation, while pore-mouth Fe@C interfacial environments promote secondary glycolysis of soluble fragments to BHET. These findings establish waste EPS as a viable carbonaceous precursor for recyclable Fe@graphitic-carbon (Fe@C) catalysts and demonstrate a dual waste-polymer valorization route for PET chemical recycling. Further studies on EG recovery, closed-loop catalyst mass retention, and isolated BHET purification will be needed to evaluate practical implementation.

## Figures and Tables

**Table 1:** Textural properties of EPS-HCP-derived carbons and Fe/carbon catalysts with Ar physisorption.

Sample	Fe Conc. (wt.%)	S_BET_ (m^2^ g^−1^)	S_micro_ (m^2^ g^−1^)	S_ext_ (m^2^ g^−1^)	V_micro_ (cm^3^ g^−1^)	V_total_ (cm^3^ g^−1^)	Pore Size (Å)	
							HK	BJH
as-EPS-HCP	-	909.6	259.4	650.2	0.077	0.830	3.60	39.81
EPS-HCP_800_	-	329.1	290.3	38.7	0.106	0.166	5.90	31.33
*1*Fe@EPS-HCP_800_	0.88	324.0	165.1	164.4	0.062	0.253	6.05	40.05
*3*Fe@EPS-HCP_800_	3.35	331.9	208.1	128.8	0.079	0.220	5.71	38.34
*5*Fe@EPS-HCP_800_	5.25	434.2	237.2	204.9	0.087	0.353	5.69	45.45
*7*Fe@EPS-HCP_800_	7.20	332.9	219.9	113.0	0.082	0.222	5.54	42.11

The Fe concentration was determined by ICP-AES analysis. S_BET_ was obtained by the BET method. S_micro_ and V_micro_ were estimated from micropore analysis. V_total_ was taken near P/P_0_ ≈ 0.99. HK and BJH pore sizes are reported in Å, and BJH values were obtained from the desorption branch.

**Table 2:** Apparent kinetic parameters for PET glycolysis over *5*Fe@EPS-HCP_800_ estimated from the Avrami–Erofeev/Weibull model, with PFO constants shown for comparison.

T (°C)	T (K)	Time Range (min)	*k*_A_ (min^−1^)	*n*	R^2^ (Avrami)	*k*_PFO_ (min^−1^)
170	443.15	0–240	9.94 × 10^−4^	1.145	0.997	7.73 × 10^−4^
180	453.15	0–240	5.61 × 10^−3^	1.615	0.996	5.10 × 10^−3^
190	463.15	0–240	1.13 × 10^−2^	1.645	0.997	1.20 × 10^−2^
200	473.15	0–120	3.99 × 10^−2^	1.543	0.999	4.66 × 10^−2^

T, reaction temperature; *k*_A_, Avrami–Erofeev/Weibull apparent rate constant; *n*, Avrami exponent; R^2^, coefficient of determination; *k*_PFO_, pseudo-first-order rate constant; and X, fractional PET conversion. Data at X = 1 were excluded from the linearized PFO analysis because −ln(1 − X) diverges, whereas Avrami–Erofeev/Weibull fitting was performed in conversion space.

**Table 3:** Arrhenius parameters calculated from temperature-dependent apparent rate constants.

Kinetic Model Used for Arrhenius Plot	ln A	A (min^−1^)	E_a_ (kJ mol^−1^)	R^2^ (Arrhenius)	Use in Manuscript
Avrami–Erofeev/Weibull	49.06	2.03 × 10^21^	205.6	0.978	Selected model
Pseudo-first-order	55.33	1.07 × 10^24^	229.6	0.982	Literature comparison only

A is an apparent pre-exponential factor for the global solid–liquid glycolysis process; therefore, it should not be assigned elementary molecular significance.

**Table 4:** Comparison with representative deep eutectic solvent and heterogeneous catalysts for PET glycolysis to BHET.

Catalyst	Catalyst Origin/Design	Representative Conditions	BHET Yield (%)	Reusability or Limitation	Ref.
*5*Fe@EPS-HCP_800_	Solid Fe@graphtic-carbon catalyst derived from waste EPS	200 °C; 2 h; PET, 2.00 g; EG, 20.00 g; 10 wt.% catalyst	94.6	10 compensated reuse runs. Fe not detected in product solution	This work
Urea/ZnCl_2_ DES	Liquid metal-containing DES	170 °C; 0.5 h; PET, 5 g; EG, 20 g; 5 wt.% catalyst	82.8	100% PET conversion. Liquid DES phase required recovery from EG and BHET	[15]
ChCl-Zn(OAc)_2_ DES	Choline chloride-based DES	180 °C; 6 h; PET, 0.2 g; EG, 1.6 g; 7 wt.% catalyst	91.6	>99% PET conversion. Liquid DES phase required recovery from EG and BHET	[16]
Fe_3_O_4_/MWCNT	Fe_3_O_4_/MWCNT	190 °C; 2 h; PET, 2.0 g; EG, 20 g; 5 wt.% catalyst	100	8 sequential runs	[19]
ZnO-Fe_3_O_4_ HMNAs	Magnetic Lewis acid	190 °C, 1.5 h; PET 10 g; EG 60 g; 1 wt.% catalyst	92.3	Magnetically recoverable. 5 cycles. Autoclave reactor	[20]
Orange peel ash	Biomass ash	190 °C, 1.5 h; PET 0.48 g; EG 2.5 g; 10.4 wt.% catalyst	79.0	Reused with catalyst make-up. Gradual activity loss was attributed to active-site leaching	[21]
Bamboo leaf ash	Si/metal-oxide ash	190 °C, 3.5 h; PET 0.48 g; EG 2.5 g; 20.8 wt.% catalyst	83.0	4 cycles. Yield decreased during reuse	[22]
Oyster shell catalyst	Ca-rich shell-waste catalyst	195 °C; PET, 5.0 g; EG 20.0 g; 1 h; 1.0 wt.% catalyst	68.6	Optimization-focused study using shell-waste catalyst	[23]
Ostrich eggshell catalyst	CaO-rich shell-waste catalyst	192 °C; PET, 5.0 g; EG 75.0 g; 2 h; 1.0 wt.% catalyst	76.0	Bio-waste catalyst	[24]
PET-bottle label-derived catalyst	Bottle-label-derived	200 °C, 1.5 h; mass ratio of EG/PET, 5; 1.0 wt.% catalyst	95.8	Deactivation after first reuse. Autoclave reactor	[25]
Banana peel ash	Potassium-rich biomass-derived catalyst	180 °C; 1.5 h; PET, 2 g; EG, 20 mL; catalyst, 0.2 g; atmospheric pressure	65.1	Simultaneous valorization of PET and banana-peel waste. Catalyst reuse was not demonstrated	[26]

The DES entries are presented as BHET yield values. The comparison includes liquid DES catalysts and solid heterogeneous catalysts. Direct numerical ranking should be made cautiously because PET source, EG/PET ratio, catalyst loading, reactor conditions, BHET quantification, and recovery protocols differ among studies.

## Data Availability

The data presented in this study are available from the corresponding authors upon reasonable request. Processed spectroscopic, sorption, kinetic, ICP, and recyclability data are summarized in the manuscript and Supporting Information.

## Supplementary Materials

The following supporting information can be downloaded at: https://www.mdpi.com/article/10.3390/polym18161983/s1, Table S1: Assignment of characteristic FT-IR absorption bands observed for pristine expanded polystyrene (EPS) and hypercrosslinked EPS (EPS-HCP); Table S2: Raman deconvolution parameters for EPS-HCP-derived carbon supports and mFe@EPS-HCP800 catalysts; Table S3: Actual Fe loading, XRD-derived apparent metallic Fe crystallite size, and Fe leaching from mFe@EPS-HCP800 catalysts after PET glycolysis; Table S4: Total basicity and acidity of mFe@EPS-HCP800 catalysts estimated from CO2-TPD and NH3-TPD. Figure S1: Structural and textural characterization of as-prepared EPS-HCP; Figure S2: XRD deconvolution analysis of the diffraction region associated with Fe-containing and graphitic phases; Figure S3: SEM images of EPS-HCP800 and mFe@EPS-HCP800 catalysts prepared with different nominal Fe loadings (m = 0, 1, 3, 5, and 7 wt.%); Figure S4: HAADF-STEM images and corresponding EDS elemental line-scan profiles of 1Fe@EPS-HCP800, 3Fe@EPS-HCP800, 5Fe@EPS-HCP800, and 7Fe@EPS-HCP800; Figure S5: Catalytic glycolysis of PET over control carbon materials and Fe-free EPS-HCP-derived carbons; Figure S6: Arrhenius plot for PET glycolysis over 5Fe@EPS-HCP800 based on the apparent Avrami-Erofeev rate constants (kA); Figure S7: Surface acid and base properties of mFe@EPS-HCP800 catalysts determined by temperature programmed desorption.

## Abbreviations

^13^C CP/MAS NMR, solid-state ^13^C cross-polarization/magic-angle-spinning nuclear magnetic resonance; as-EPS-HCP, as-prepared EPS-derived hypercrosslinked polymer; BET, Brunauer–Emmett–Teller; BHET, bis(2-hydroxyethyl) terephthalate; CNT, carbon nanotube; DTG, derivative thermogravimetry; EDS, energy-dispersive X-ray spectroscopy; EG, ethylene glycol; EPS, expanded polystyrene; Fe@C, Fe@graphitic-carbon; FT-IR, Fourier-transform infrared spectroscopy; HAADF-STEM, high-angle annular dark-field scanning transmission electron microscopy; HCP, hypercrosslinked polymer; HK, Horvath-Kawazoe; HPLC, high-performance liquid chromatography; HRTEM, high-resolution transmission electron microscopy; ICP-AES, inductively coupled plasma atomic emission spectroscopy; MWCNTs, multiwalled carbon nanotubes; PET, poly(ethylene terephthalate); PFO, pseudo-first-order; SEM, scanning electron microscopy; TEM, transmission electron microscopy; TPD, temperature-programmed desorption; TGA, thermogravimetric analysis; THF, tetrahydrofuran; XPS, X-ray photoelectron spectroscopy; XRD, X-ray diffraction.
