# Supplementary material for: Upcycling Waste Expanded Polystyrene into Fe@Graphitic-Carbon Catalysts for Glycolytic Recycling of PET to BHET
Source: Polymers (Basel). 2026 Aug 14;18(16):1983. doi: 10.3390/polym18161983 (PMC13517327; doi:10.3390/polym18161983)
Supplement: Supplementary file 1 [file polymers-18-01983-s001.zip › polymers-4470372-supplementary.pdf]

Supporting Information

Upcycling Waste Expanded Polystyrene into Fe@Graphitic-Carbon Catalysts for Glycolytic Recycling of PET to BHET

Jong In Choi [1,2,†], Chitra Sarkar [1,†], Yoojin Kang [1,2], Saira Kanwal [1,3], Youn-Sang Bae [2,*] and Do-Young Hong [1,3,*]

1 Research Center for Nanocatalysts, Korea Research Institute of Chemical Technology (KRICT), 141 Gajeong-ro, Yuseong, Daejeon 34114, Republic of Korea
2 Department of Chemical and Biomolecular Engineering, Yonsei University, 50 Yonsei-ro, Seodaemun-gu, Seoul 03722, Republic of Korea; mowbae@yonsei.ac.kr
3 Department of Advanced Materials and Chemical Engineering, University of Science and Technology (UST), Yuseong-gu, Daejeon 34113, Republic of Korea; dyhong@krict.re.kr
* Correspondence: mowbae@yonsei.ac.kr (Y.-S.B.); dyhong@krict.re.kr (D.-Y.H.)
† These authors contributed equally to this work.

Table S1. Assignment of characteristic FT-IR absorption bands observed for pristine expanded polystyrene (EPS) and hypercrosslinked EPS (EPS-HCP). The listed bands were used to verify the aromatic polystyrene framework, methylene-bridged hypercrosslinked network, and residual oxygen-/chlorine-containing functionalities introduced during the formaldehyde dimethyl acetal/1,2-dichloroethane-assisted hypercrosslinking step.

Structural group	Wavenumber (cm^{-1})
Aromatic C–H stretching	3025, 3058, 3083
Aliphatic Symmetric/Asymmetric C–H stretching	2848, 2918
C=O stretching	1697
C=C stretching	1445, 1492, 1598
C–O–C stretching	1095, 1767
C–Cl vibrations	1266
C–H bending vibrations in multi-substituted benzene ring	818
C–H bending vibrations in monosubstituted benzene ring	697, 755
C–H deformation vibrations in monosubstituted benzene ring	540

Table S2. Raman deconvolution parameters for EPS-HCP-derived carbon supports and *m*Fe@EPS-HCP_{800} catalysts. The I_D/I_G ratio describes the relative contribution of disordered and graphitic carbon domains, while the $I_{D''}/I_G$ ratio is used as a comparative descriptor for amorphous carbon contributions after carbonization and Fe-assisted graphitic reconstruction.

Samples	I_D/I_G	$I_{D''}/I_G$
EPS-HCP_{700}	1.05	0.46
EPS-HCP_{800}	0.97	0.47
EPS-HCP_{900}	0.88	0.41
*1*Fe@EPS-HCP_{800}	1.00	0.39
*3*Fe@EPS-HCP_{800}	0.99	0.38
*5*Fe@EPS-HCP_{800}	0.98	0.34
*7*Fe@EPS-HCP_{800}	0.97	0.44

Note: *The D″/G intensity ratio is used here as a comparative descriptor of the amorphous carbon contribution. The lower value for 5Fe@EPS-HCP_{800} indicates more effective Fe-assisted graphitic reconstruction than the 7 wt.% Fe sample, for which excessive Fe loading can promote particle growth and less uniform carbon reconstruction.*

Table S3. Actual Fe loading, XRD-derived apparent metallic Fe crystallite size, and Fe leaching from *m*Fe@EPS-HCP_{800} catalysts after PET glycolysis. The crystallite-size trend was used to evaluate Fe-particle growth with increasing Fe loading, while ICP-AES analysis of the product solution was used to assess the stability of Fe encapsulation within the graphitic carbon matrix.

Catalyst	Actual Fe loading (wt.%)	Apparent metallic Fe crystallite size from XRD (nm)	Fe in product solution by ICP-AES (mg kg^{-1})
*1*Fe@EPS-HCP_{800}	0.88	20.6	Not detected
*3*Fe@EPS-HCP_{800}	3.35	34.6	Not detected
*5*Fe@EPS-HCP_{800}	5.25	34.9	Not detected
*7*Fe@EPS-HCP_{800}	7.20	61.7	9.1

Note: *Crystallite sizes were estimated from the metallic Fe-like XRD reflection using the Scherrer equation and should be interpreted as comparative apparent values. "Not detected" indicates that the Fe concentration was below the analytical detection limit (<1 mg kg^{-1}) of the ICP-AES analysis performed on the post-reaction product solution.*

Table S4. Total basicity and acidity of *m*Fe@EPS-HCP$_{800}$ catalysts estimated from CO_2-TPD and NH_3-TPD.

	Total basicity (mmol/g)	Total acidity (mmol/g)
*1*Fe@EPS-HCP$_{800}$	0.26	2.25
*3*Fe@EPS-HCP$_{800}$	0.30	2.20
*5*Fe@EPS-HCP$_{800}$	0.31	2.53
*7*Fe@EPS-HCP$_{800}$	0.26	2.39

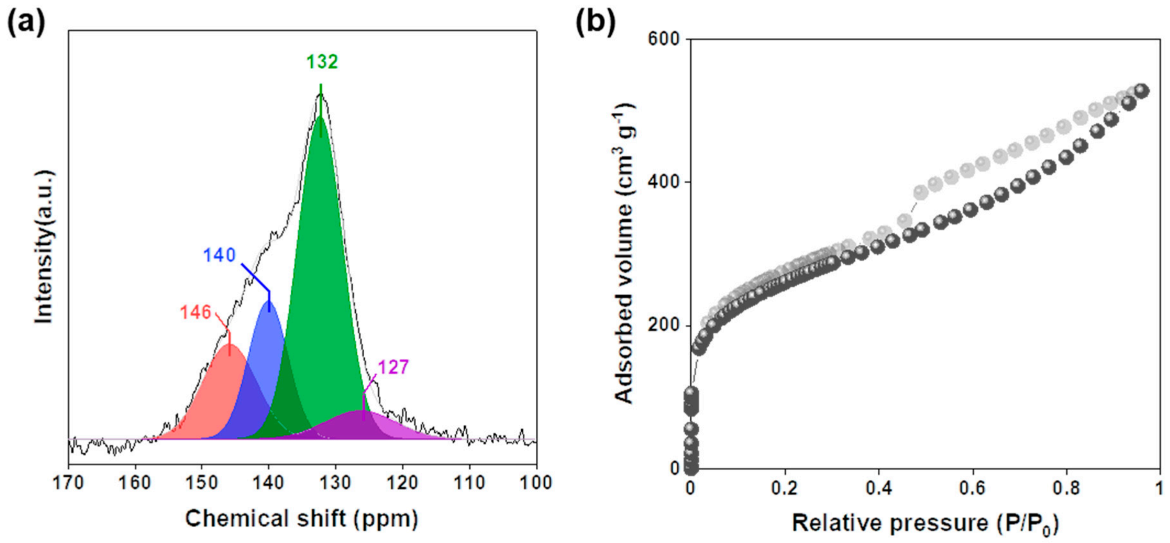

Figure S1. Structural and textural characterization of as-prepared EPS-HCP. (a) Deconvoluted solid-state ^{13}C CP/MAS NMR spectrum showing aromatic carbon environments and bridge-related carbon environments generated during hypercrosslinking. (b) N_2 adsorption–desorption isotherm of as-EPS-HCP, confirming the formation of a porous hypercrosslinked polymer network before carbonization.

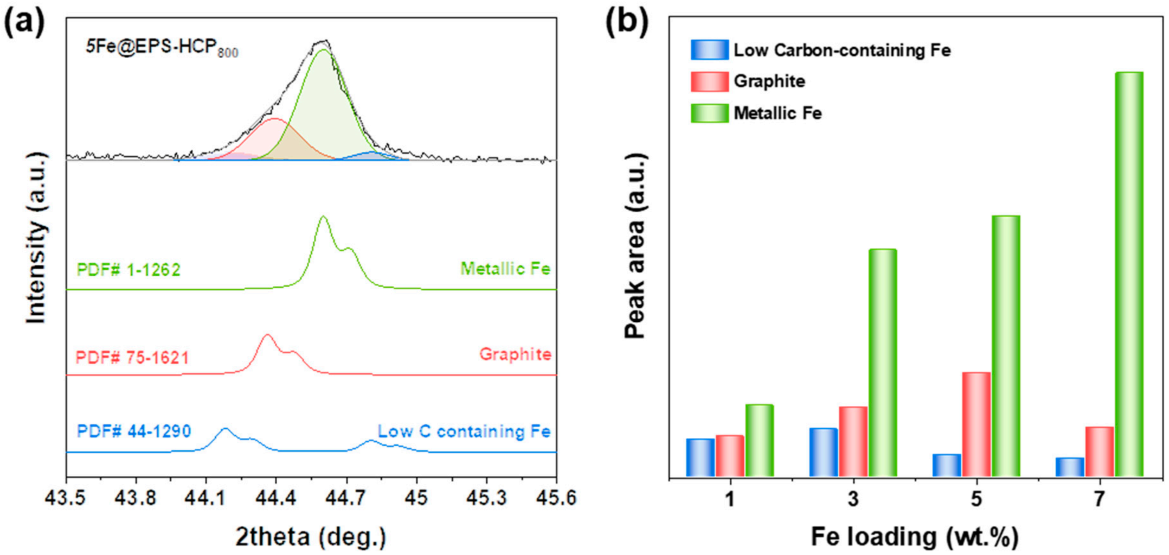

Figure S2. XRD deconvolution analysis of the diffraction region associated with Fe-containing and graphitic phases. (a) Reference diffraction patterns of metallic Fe, graphite, and low-carbon-containing Fe compared with the deconvoluted diffraction profile of $5Fe@EPS-HCP_{800}$. (b) Relative peak areas obtained by deconvolution in the 2theta range of 43.8-45.0°, showing the Fe-loading-dependent contribution from metallic Fe, graphite-like carbon, and low-carbon-containing Fe-related components.

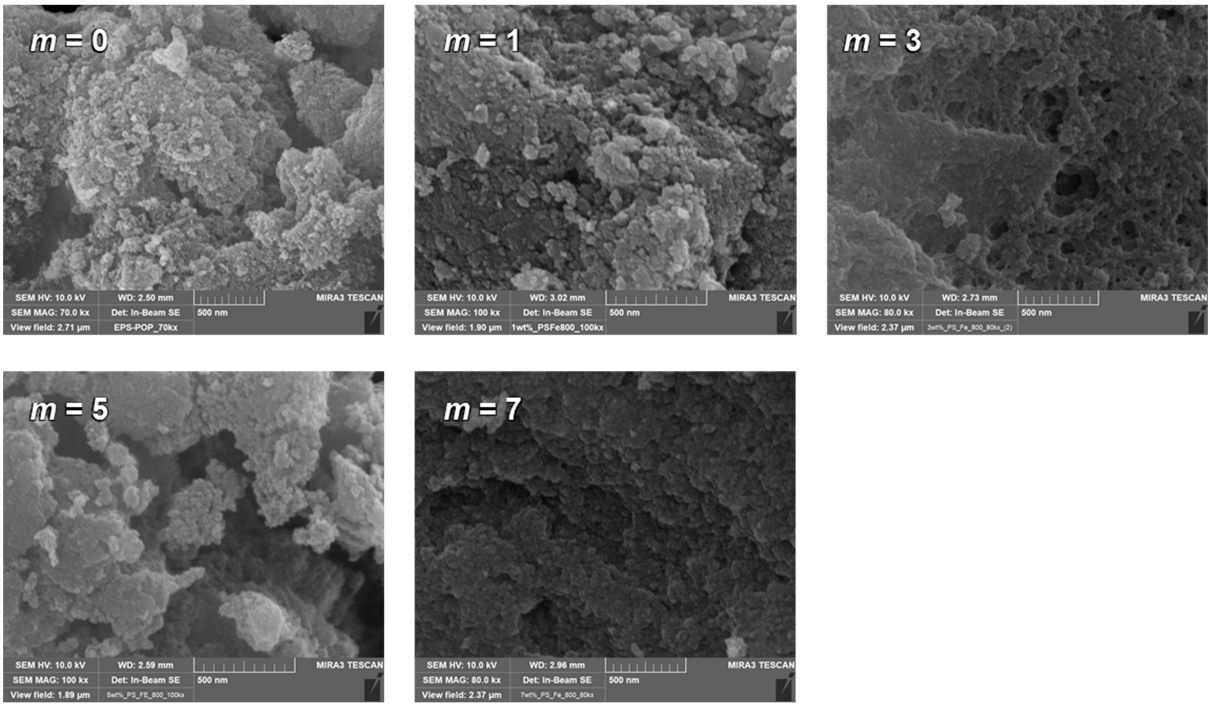

Figure S3. SEM images of $EPS-HCP_{800}$ and $m$$Fe@EPS-HCP_{800}$ catalysts prepared with different nominal Fe loadings (m = 0, 1, 3, 5, and 7 wt.%). The images compare the carbon-particle morphology and surface texture after Fe-assisted carbonization.

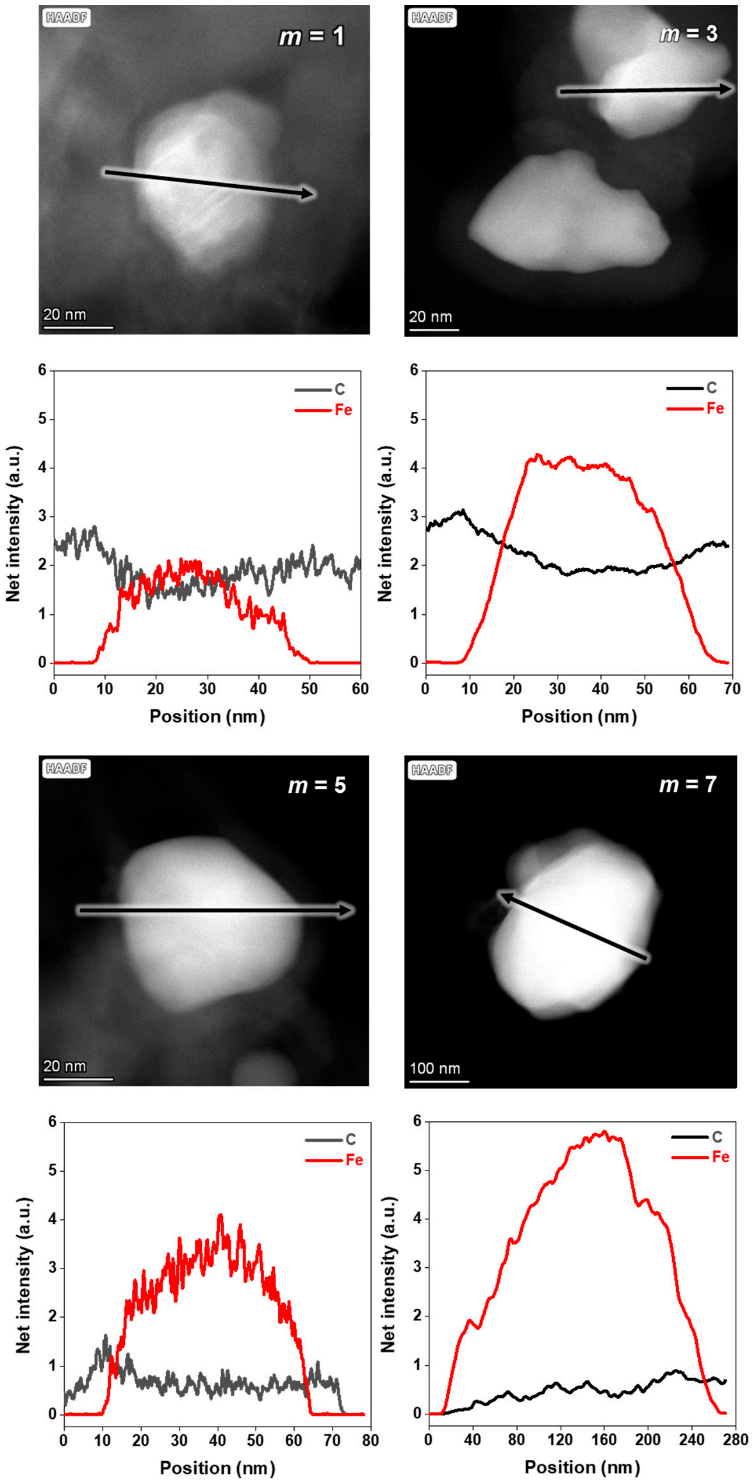

Figure S4. HAADF-STEM images and corresponding EDS elemental line-scan profiles of *1*Fe@EPS-HCP$_{800}$, *3*Fe@EPS-HCP$_{800}$, *5*Fe@EPS-HCP$_{800}$, and *7*Fe@EPS-HCP$_{800}$. Arrows indicate the scan directions, and the gray and red profiles represent C and Fe signals, respectively. The 1-5 wt.% Fe catalysts show Fe-rich domains accompanied by carbon signals on both sides, consistent with carbon encapsulation of Fe-rich domains. The 7 wt.% Fe catalyst shows a broader Fe profile and a metal-rich side without a corresponding carbon signal, indicating incomplete graphitic coverage or partial Fe exposure. This structural difference is consistent with the Fe leaching detected only for *7*Fe@EPS-HCP$_{800}$.

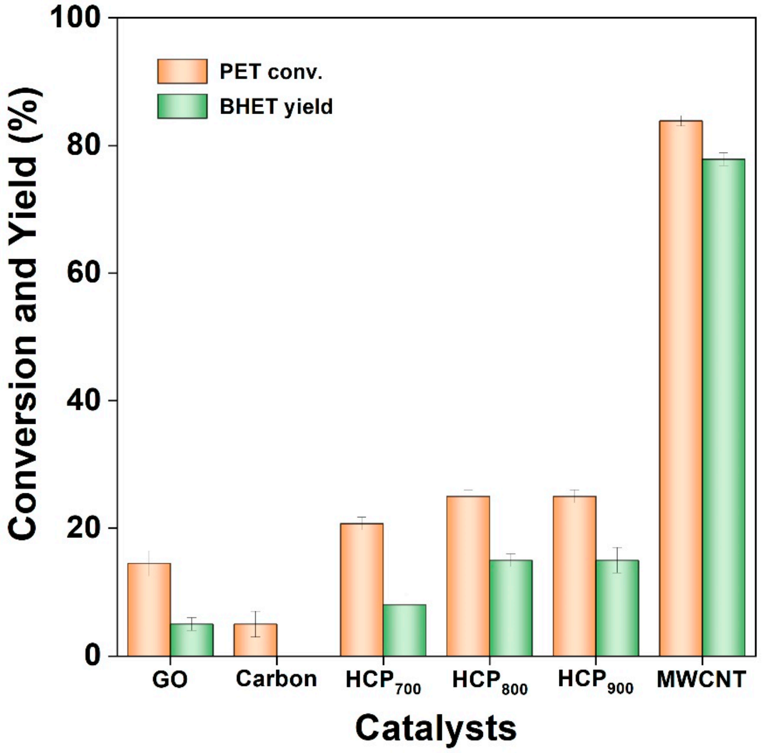

Figure S5. Catalytic glycolysis of PET over control carbon materials and Fe-free EPS-HCP-derived carbons. PET conversion and BHET yield were compared over graphene oxide (GO), amorphous carbon, EPS-HCP-derived carbons carbonized at 700, 800, and 900 °C, and commercial MWCNTs. Reaction conditions: PET, 2.00 g; ethylene glycol, 20.00 g; catalyst, 0.20 g; 200 °C; 2 h.

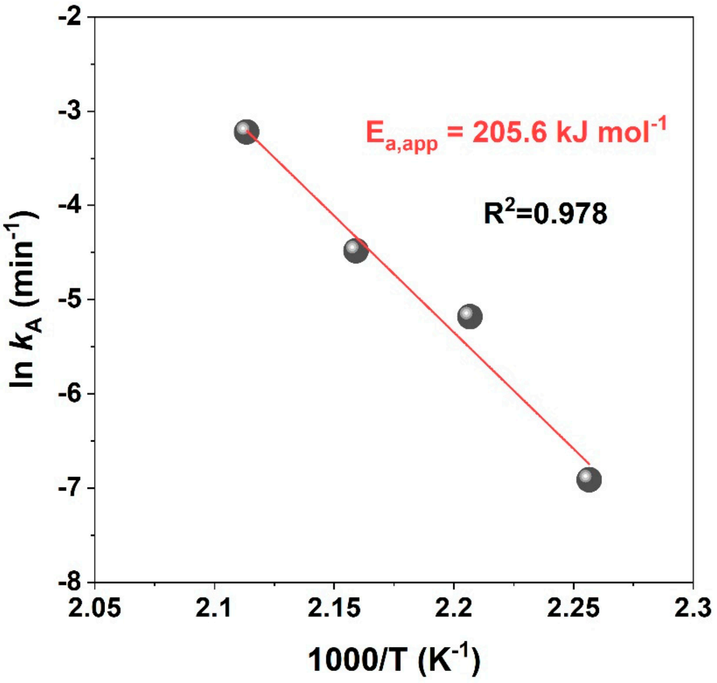

Figure S6. Arrhenius plot for PET glycolysis over 5Fe@EPS-HCP$_{800}$ based on the apparent Avrami-Erofeev rate constants (k_A). The apparent activation energy was calculated from the slope of ln k_A versus 1/T and is used as a comparative descriptor for the heterogeneous PET depolymerization process.

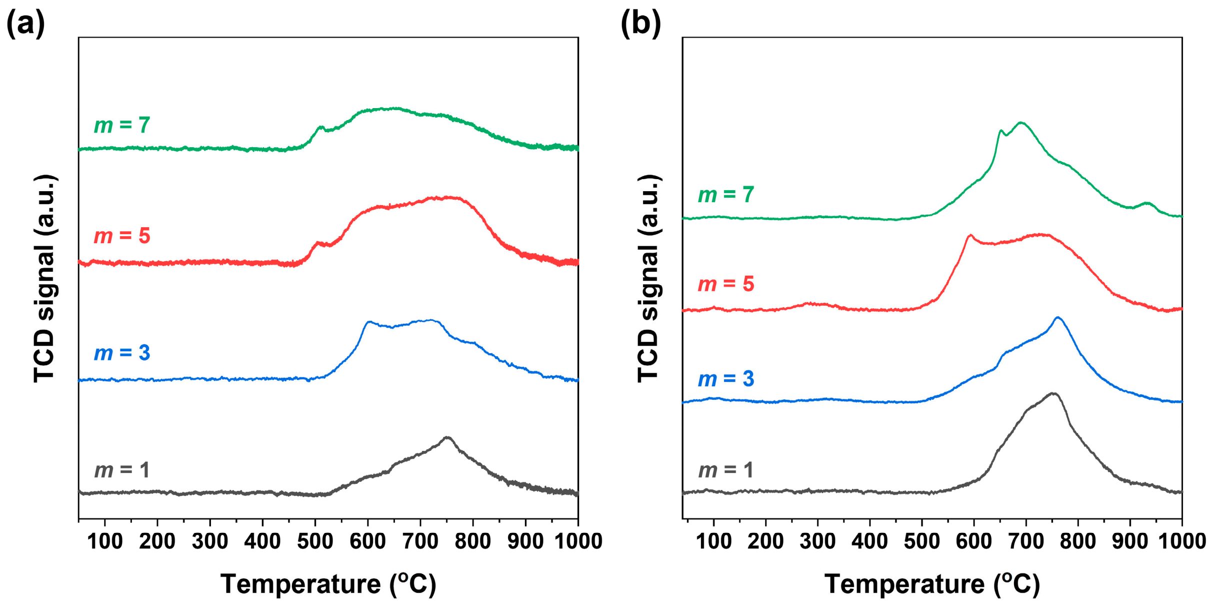

Figure S7. Surface acid and base properties of *m*Fe@EPS-HCP$_{800}$ catalysts determined by temperature-programmed desorption. (a) CO_2-TPD profiles and (b) NH_3-TPD profiles of catalysts with nominal Fe loadings of *m* = 1, 3, 5, and 7 wt.%.
